# Digital Health Strategies for Cervical Cancer Control in Low- and Middle-Income Countries: Systematic Review of Current Implementations and Gaps in Research

**DOI:** 10.2196/23350

**Published:** 2021-05-27

**Authors:** Andrea H Rossman, Hadley W Reid, Michelle M Pieters, Cecelia Mizelle, Megan von Isenburg, Nimmi Ramanujam, Megan J Huchko, Lavanya Vasudevan

**Affiliations:** 1 Department of Biomedical Engineering Pratt School of Engineering Duke University Durham, NC United States; 2 Duke School of Medicine Durham, NC United States; 3 Duke Global Health Institute Durham, NC United States; 4 Medical Center Library Duke University Durham, NC United States; 5 Department of Obstetrics and Gynecology Duke University School of Medicine Durham, NC United States; 6 Department of Family Medicine and Community Health Duke University School of Medicine Durham, NC United States

**Keywords:** cervical cancer, digital health, mobile phones, low- and middle-income countries, colposcopy, uterine cervical neoplasms, telemedicine or mobile apps, cell phones, developing countries

## Abstract

**Background:**

Nearly 90% of deaths due to cervical cancer occur in low- and middle-income countries (LMICs). In recent years, many digital health strategies have been implemented in LMICs to ameliorate patient-, provider-, and health system–level challenges in cervical cancer control. However, there are limited efforts to systematically review the effectiveness and current landscape of digital health strategies for cervical cancer control in LMICs.

**Objective:**

We aim to conduct a systematic review of digital health strategies for cervical cancer control in LMICs to assess their effectiveness, describe the range of strategies used, and summarize challenges in their implementation.

**Methods:**

A systematic search was conducted to identify publications describing digital health strategies for cervical cancer control in LMICs from 5 academic databases and Google Scholar. The review excluded digital strategies associated with improving vaccination coverage against human papillomavirus. Titles and abstracts were screened, and full texts were reviewed for eligibility. A structured data extraction template was used to summarize the information from the included studies. The risk of bias and data reporting guidelines for mobile health were assessed for each study. A meta-analysis of effectiveness was planned along with a narrative review of digital health strategies, implementation challenges, and opportunities for future research.

**Results:**

In the 27 included studies, interventions for cervical cancer control focused on secondary prevention (ie, screening and treatment of precancerous lesions) and digital health strategies to facilitate patient education, digital cervicography, health worker training, and data quality. Most of the included studies were conducted in sub-Saharan Africa, with fewer studies in other LMIC settings in Asia or South America. A low risk of bias was found in 2 studies, and a moderate risk of bias was found in 4 studies, while the remaining 21 studies had a high risk of bias. A meta-analysis of effectiveness was not conducted because of insufficient studies with robust study designs and matched outcomes or interventions.

**Conclusions:**

Current evidence on the effectiveness of digital health strategies for cervical cancer control is limited and, in most cases, is associated with a high risk of bias. Further studies are recommended to expand the investigation of digital health strategies for cervical cancer using robust study designs, explore other LMIC settings with a high burden of cervical cancer (eg, South America), and test a greater diversity of digital strategies.

## Introduction

### Background

Annually, 311,000 women die from cervical cancer worldwide, with 90% of the deaths occurring in low- and middle-income countries (LMICs). [1]. To reduce this high burden of mortality, it is critical to implement and scale sustainable and effective cervical cancer control programs in LMICs. However, cervical cancer control programs in LMICs must overcome individual-, provider-, and health system–level bottlenecks to health service delivery, access, and utilization [2-5]. In the last decade, health systems in LMICs have taken advantage of the increasing prevalence of digital technologies, particularly mobile phones, to circumvent some of the bottlenecks in cervical cancer control. However, systematic reviews of the effectiveness of such digital health strategies in LMIC settings are lacking, especially regarding how such strategies may improve the delivery, access, and utilization of cervical cancer control programs [4].

Primary prevention strategies for cervical cancer control focus on vaccination against human papillomavirus (HPV), whereas secondary prevention strategies focus on early screening and treatment of precancerous lesions [6,7]. Where implementation of preventive services is inadequate, cervical cancers are typically detected at advanced stages, further contributing to high mortality rates [8,9]. Commonly used screening methods include visual inspection with acetic acid (VIA), visual inspection with Lugol iodine, and HPV DNA testing [10,11]. Although an initial cervical cancer screening is possible at primary health facilities, or even at the community level by frontline health workers, follow-up procedures, diagnosis, and treatment require access to trained medical providers (eg, expert colposcopists). The treatment of invasive cancer also requires secondary or tertiary health facilities with specialized equipment for surgery, chemotherapy, and radiation [11]. The management of invasive cancer includes long-term retention of women in care, with regular follow-ups and palliative care as applicable. To be effective, cervical cancer control programs must not only achieve high rates of screening coverage among eligible women but also ensure that women who screen positive receive timely treatment and support for long-term management [12]. There is a lack of systematic reviews to understand how digital health strategies affect cervical cancer control in LMICs across the continuum spanning from prevention to palliative care.

There is growing interest from local, national, and global stakeholders in integrating digital health strategies with cervical cancer control programs in LMICs. For instance, the Global Action Plan for the Prevention and Control of Noncommunicable Diseases 2013-2020 highlights the use of digital health strategies for health education, promotion, and communication, especially in populations with low literacy and health awareness [13]. Examples of using digital health for cervical cancer control include the World Health Organization and the International Telecommunications Union’s *Be he@lthy, Be mobile* initiative, which launched a mobile phone text messaging campaign to improve the awareness of cervical cancer screening in Zambia [14]. In another instance, the Ministry of Health in Peru successfully completed a national pilot program of using text messages to notify women about their HPV screening results [15]. In light of the expanding local, national, and global efforts, evidence of successful implementation and impact is needed to drive further research on and development of digital strategies for cervical cancer control in LMICs.

Currently, much of the published literature describing the use of digital strategies for cervical cancer control comes from high-income settings [16-19]. However, a previous systematic review examined the effectiveness of digital strategies for cervical cancer control in LMICs [20]. In this review, authors identified 8 eligible studies, most of which lacked rigorous study designs and evidence. With the increasing use of digital health strategies in LMICs, an updated review is necessary to evaluate the contextual effectiveness of such strategies for cervical cancer control as compared with high-income settings. Furthermore, the synthesis of key implementation challenges and opportunities for future research is needed to prioritize and increase the success of digital health strategies for cervical cancer control in LMICs.

### Objectives

The primary objectives of the review were to assess the effects of using mobile devices on the following:

- Facilitate task shifting for cervical cancer screening, treatment of precancerous lesions, and management of LMICs as compared with usual care.
- Reduce delays in postscreening treatment initiation among women in LMICs as compared with usual care.
- Assess and improve cervical cancer knowledge or awareness among women in LMICs as compared with usual care.

The secondary objectives were to describe the following:

- Digital health strategies used for cervical cancer control, including those used to visualize the cervix using a mobile device at the point of care in LMICs.
- Challenges associated with the implementation of digital health strategies for cervical cancer control in LMICs.

## Methods

### Protocol and Registration

The review protocol was registered with the PROSPERO database for prospectively registered systematic reviews (protocol #CRD42017071560). Deviations from the registered protocol and unused methods are included in Multimedia Appendix 1. The findings of this systematic review are reported in accordance with the PRISMA (Preferred Reporting Items for Systematic Reviews and Meta-Analyses) checklist and are specified in Multimedia Appendix 2 [21].

### Eligibility Criteria

#### Participants

For the assessment of the primary and secondary review objectives, we included studies of all cadres of health care workers (eg, medical doctors, nurses, midwives, community health workers) providing any cervical cancer screening, treatment, and management services, as well as women (of all ages) receiving any cervical cancer education, screening, treatment, and management services.

#### Interventions

For the assessment of primary and secondary review objectives, we included any intervention wherein mobile devices were used in the screening, treatment, and management of cervical cancer. These included studies in which mobile devices were used to do the following:

- Facilitate the visualization of the cervix (cervicography).
- Facilitate communication between health care providers for diagnosis, consultation, or referral.
- Provide training or support to health care providers, especially in the context of task shifting.
- Communicate with patients to provide appointment reminders, test results, disease progression monitoring, coordinate services, etc.
- Improve cervical cancer knowledge or awareness among women.
- Facilitate cervical cancer screening, treatment of preinvasive lesions or cervical cancer management.

We excluded the following types of studies:

- Where the intervention used a nonmobile phone device (eg, digital camera, television) to visualize the cervix.
- Where the intervention exclusively focused on the use of mobile devices for providing education about HPV vaccination or to improve its coverage, because these activities generally fall within the purview of immunization programs. In addition, other published systematic reviews have examined the efficacy of mobile phone interventions for HPV vaccination [22-24].
- Where the intervention was only described or conceptualized without actual implementation or evaluation.
- Where abstracts were only published in languages other than English and for which published English translations were not available.
- Where full texts were not available.
- Where the publications were reviews, systematic reviews, conference proceedings, blogs, reports, or other nonpeer-reviewed sources.

#### Comparators

Details of the comparator group were extracted from the studies and included in the analysis of primary and secondary review objectives. The comparison was usual care, which could include the use of traditional colposcopy and nondigital, paper-based strategies for data collection, communication, and dissemination in cervical cancer control programs.

#### Outcomes

For the review of the primary review objectives, the outcomes of interest were as follows:

1. Coverage and timeliness of cervical cancer screening, treatment, and management.
2. Cervical cancer knowledge or competency (ie, training) of health worker.
3. Cervical cancer knowledge or awareness among women.

Outcome data were not reviewed quantitatively because of the descriptive nature of the secondary review objectives.

#### Study Design

We included randomized and nonrandomized controlled study designs (controlled before-and-after studies with at least 2 intervention sites or interrupted time-series studies) in the qualitative synthesis and meta-analysis of the primary review objectives. We included all study designs for a narrative review of the secondary review objectives.

#### Settings

For both the primary and secondary review objectives, we included studies from any country listed as low or middle income according to the World Bank Group classification [25].

### Information Sources

#### Electronic Searches

The following 5 electronic databases were searched for studies published in English: PubMed, Embase, Web of Science, Scopus, and CINAHL. We included studies from January 1, 1992, to September 19, 2020 (date of search), with 1992 being the year the first commercial text message was sent.

#### Search Strategy

A systematic search strategy (Multimedia Appendix 3) was developed, including a detailed search string comprising terms from 3 broad categories, namely, digital health, cervical cancer, and LMICs. The search terms were customized for each of the 5 electronic databases. A Google search was conducted with an abbreviated search string, as described in Multimedia Appendix 3, and the first 100 search results were analyzed.

### Study Selection

Search records were imported into the reference management software and duplicates were removed. The titles and abstracts of the records were screened according to the predefined eligibility criteria. For each record, 2 reviewers discussed and resolved any ambiguities in screening outcomes during abstract and full-text screening. Interrater reliability was assessed by 2 reviewers independently screening a 10% sample of sources for inclusion based on abstract evaluation. A Cohen κ value of 0.6 was predetermined as an acceptable interrater agreement.

### Data Collection Process and Items

We used a structured template, which was adapted based on the template from Cochrane, to extract relevant data from each included study [26]. Primary data items extracted using the template included study location, sample size, population, study duration and design, cervical cancer service or procedure, the description of digital health intervention, conclusions, study limitations, and information for assessing the risk of bias. In addition, quantitative outcome data were extracted from the studies included in the review of the primary objectives.

### Risk of Bias in Individual Studies

We assessed the risk of bias for all studies using the Effective Public Health Practice Project’s Quality Assessment Tool for Quantitative Studies [27].

### Additional Analyses

We assessed the quality of data reporting for each included study using the mobile health (mHealth) evidence reporting and assessment (mERA) checklist [28]. This 16-item checklist aims to enhance the replicability of mHealth interventions by promoting the complete reporting of content, context of implementation, and technical features in peer-reviewed publications.

## Results

### Study Selection

**Figure 1:**
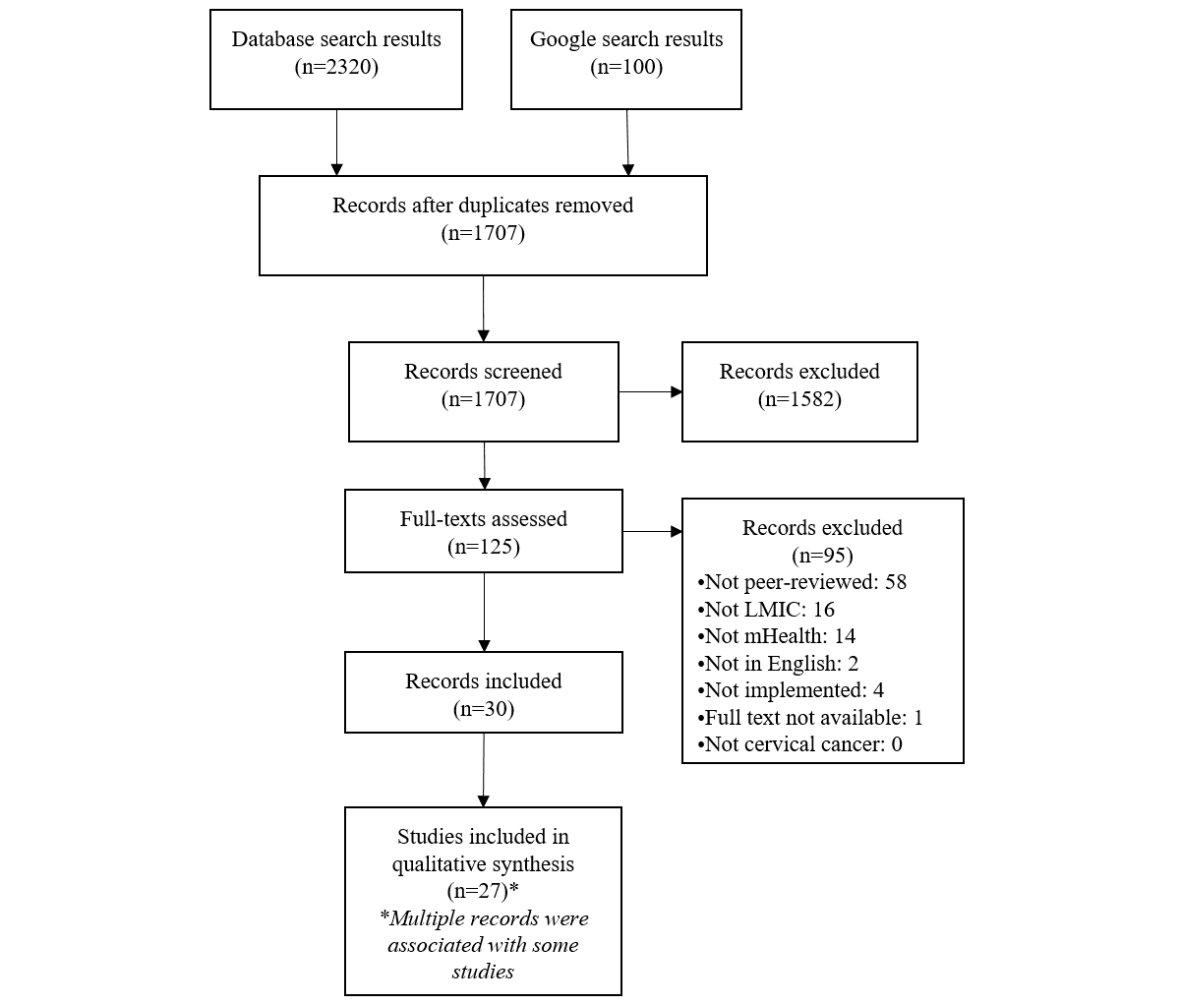
PRISMA (Preferred Reporting Items for Systematic Reviews and Meta-Analyses) flowchart of systematic search results. LMIC: low- and middle-income country; mHealth: mobile health.

Figure 1 shows the results of the study selection process. The systematic search yielded 1707 nonduplicate studies. The overall interrater agreement for screening a 10% sample was 98.8%, which corresponded to a Cohen κ value of 0.76 [29]. Following the abstract screening, 125 records were evaluated using their full text and 95 of those were excluded. Records were excluded after full-text review mainly because they did not describe a peer-reviewed study or because the intervention was conducted in a non-LMIC setting. In total, 30 records corresponding to 27 unique studies [30-59] were included in the final review.

### Study Characteristics

Characteristics of included studies are summarized in Multimedia Appendix 4 [30-59].

#### Study Design

In total, 4 included studies were randomized controlled trials (RCTs) [38,40,47-49,59]. These studies met the criteria for inclusion in the review of the primary objectives, that is, the effect of digital health strategies on cervical cancer control. A fifth study by Lima et al [41] used random allocation but was excluded from the review of the primary objectives because of the lack of a control group. Huchko et al [39] reported findings of a mixed methods analysis from a cluster RCT; however, the assignment of the digital health strategy was not randomized; thus, it did not meet the criteria for inclusion in the review of primary objectives. All 27 studies were included in the review of the secondary objectives, that is, to describe the digital health strategies used for cervical cancer control in LMICs and the challenges associated with their implementation. Nonrandomized studies used quasi-experimental [32-34,41], cross-sectional [30,31,35-37,46,50,51,53,56,57], or other [52,54,55] study designs. In total, 4 studies [42-45] described program implementation.

#### Participants

The studies included 23,393 women and 152 health care workers. Types of health care workers included clinicians (eg, gynecologists, colposcopists, and assistant medical officers), facility-based nurses, and community health nurses.

#### Intervention

Many interventions targeted health workers and implemented the use of mobile phones for digital cervicography (ie, imaging of the cervix) or for digital patient data collection [30-32,35-37,42-46,50,51,53-57]. Interventions targeting women were focused on building knowledge or awareness about cervical cancer, delivering reminders to promote the uptake of cervical cancer services or for the notification of test results [33,34,38-41,47-49,52,58,59].

#### Comparators

Most of the included studies lacked a parallel control group. Apart from the 4 RCTs and the cluster randomized trial, the only other studies with a control group were Caster et al and Romli et al [34,58].

#### Outcomes

Studies using digital cervicography primarily reported diagnostic reliability outcomes such as interrater agreement and sensitivity or specificity as compared with a gold standard diagnostic test such as histology [30,31,35,46,50,51,53-57]. Among the 4 RCTs included in the review of primary objectives, 1 study [40] measured improvement in patient knowledge and all 4 [38,40,47-49,59] measured the uptake of cervical cancer services postintervention. One RCT study [49] included a cost-effectiveness analysis. For the secondary review objectives, all 27 studies included a description of the digital health strategy used and implementation challenges.

#### Settings

The review focused on LMICs: 19 studies were conducted in sub-Saharan Africa [30,31,34-39,42-46,50,52,54-56,59], 7 in Asia [32,33,40,47-49,51,53,58], and 1 in South America [41].

### Risk of Bias Within Studies

The analysis results of the risk of bias within the studies are presented in Table 1 (Effective Public Health Practice Project Quality Assessment tool for quantitative studies). Most of the studies (21/27, 78%) were assessed to have a high risk of bias. A total of 2 studies (Erwin et al [38] and Romli et al [58]) were assessed to have a low risk of bias, whereas 4 other studies [34,40,53,59] were assessed to have a moderate risk of bias.

**Table 1:** Risk of bias assessment for included studies using the Effective Public Health Practice Project’s quality assessment tool for quantitative studies^a^.

Study	Selection bias	Study design	Confounders	Blinding	Data collection method	Withdrawals and dropouts	Global rating
Asgary et al (2016) [30]; Asgary et al (2019) [31]	1	3	3	2	1	1	3
Bhatt et al (2018) [32]	3	3	3	3	3	3	3
Caster et al (2015) [34]	1	2	3	2	2	1	2
Catarino et al (2015) [35]	2	3	3	2	1	N/A^b,c^	3
Devi et al (2018) [33]	3	3	3	3	2	3	3
Erwin et al (2019) [38]	1	1	2	2	2	1	1
Gallay et al (2017) [36]	2	3	1	2	3	1	3
Huchko et al (2019) [39]	1	3	2	3	2	2	3
Khademolhosseini et al (2017) [40]	2	1	1	3	1	1	2
Lima et al (2017) [41]	2	1	1	1	3	3	3
Linde et al (2020) [59]	1	1	1	2	3	1	2
Littman-Quinn et al (2013) [42]	2	3	3	3	3	3	3
Ndlovu et al (2014) [43]	2	3	3	3	1	3	3
Parham et al (2010) [44]	2	3	3	3	3	3	3
Peterson et al (2016) [45]	1	3	1	3	3	N/A	3
Quercia et al (2018) [37]	2	3	N/A	3	1	1	3
Quinley et al (2011) [46]	1	3	3	2	1	N/A	3
Rashid et al (2013) [47]; Rashid and Dahlui (2013) [48]; Rashid et al (2014) [49]	1	1	3	2	3	N/A	3
Ricard-Gauthier et al (2015) [50]	2	3	3	2	1	2	3
Romli et al (2020) [58]	1	1	1	2	1	1	1
Sharma et al (2018) [51]	2	3	N/A	3	2	2	3
Swanson et al (2018) [52]	1	2	1	3	3	3	3
Taghavi et al (2018) [53]	1	3	N/A	2	1	1	2
Tran et al (2018) [54]	2	3	3	2	3	3	3
Urner et al (2017) [55]	2	3	1	2	2	3	3
Yeates et al (2016) [56]	2	3	3	3	3	3	3
Yeates et al (2020) [57]	2	3	3	2	3	2	3

^a^Scores of 1, 2, and 3 indicate low, moderate, and high risks of bias, respectively. The risk of bias was assessed cumulatively for studies with multiple sources, for example, Asgary et al [30,31].

^b^N/A: not applicable.

^c^Criteria were not applicable based on a skip pattern in the Effective Public Health Practice Project tool.

### Results of Individual Studies

The study outcomes included in the review of the primary objectives are presented in Table 2.

**Table 2:** Outcomes of randomized controlled trial studies included in the review of primary objectives.

Study and participants			Outcome		Result		Summary of risk of bias^a^
**Erwin et al (2019) [38], N=851 women**							
	281 controls 272 SMS messaging 298 SMS messaging+e-voucher	Cervical cancer screening attendance within 60 days of randomization (combined for women from rural and urban settings)		Women in the SMS messaging group had 3.0 higher adjusted odds of attendance as compared with women in the control group Women in the SMS messaging+e-voucher group had 4.7 higher adjusted odds of attendance as compared with women in the control group Women in the SMS messaging+e-voucher group had 1.5 times higher adjusted odds of attendance compared with women in the SMS messaging group		The overall risk of bias was assessed to be low	
**Khademolhosseini et al (2017) [40], N=95 women**							
	47 control 48 intervention	Mean difference in pre- and posttraining knowledge among women in the intervention group as compared with those in the control group measured immediately and 3 months after SMS messaging–based training		Women in the intervention group had a mean increase in the knowledge of 8.18 points from baseline as compared with a mean increase of 0.27 points from baseline in the control group immediately posttraining Women in the intervention group had a mean increase in the knowledge of 8.35 points from baseline as compared with a mean increase of 0.17 points in the control group at 3 months of posttraining		The overall risk of bias was assessed to be moderate	
	47 control 48 intervention	Uptake of Pap test within 3 months of training in the intervention group compared with control group		At 3 months of posttraining, only 4 (5.8%) participants of the control group as compared with 23 (47.9%) participants of the intervention group had received a Pap test		The overall risk of bias was assessed to be moderate	
**Linde et al (2020) [59], N=705 women**							
	347 standard of care (control) 358 standard of care+text message	The attendance rate of follow-up cervical cancer screening among HPV^b^-positive women		Compared with standard of care, a written appointment card, the addition of one-way text messages had no effect on follow-up cervical cancer screening among HPV-positive women		The overall risk of bias was assessed to be moderate	
**Rashid and Dahlui (2013) [48], N=1000 women**							
	250 letters 250 registered letters 250 SMS messaging 250 phone calls	The uptake of Pap test in response to recall through phone call as compared with recall by letter		Compared with women receiving recall by letter, those receiving recall by phone call had 2.38 times higher odds of receiving a Pap smear		The overall risk of bias was assessed to be high	
	250 letters 250 registered letters 250 SMS messaging 250 phone calls	The uptake of Pap test in response to recall through SMS messaging as compared with recall by letter		Compared with women receiving recall by letter, those receiving recall by SMS messaging had no significant change in the odds of receiving a Pap smear		The overall risk of bias was assessed to be high	

^a^Assessed using the Effective Public Health Practice Project risk of bias assessment tool for quantitative studies.

^b^HPV: human papillomavirus.

### Synthesis of Results

Findings From the Review of Primary Objectives: Effectiveness of Digital Health Strategies for Cervical Cancer Control

We did not conduct a meta-analysis of the included studies because of variations in the nature of the interventions, outcome measures, and small number of studies included in the primary review of the effect of mobile devices. The only included study with a low risk of bias found that SMS behavior change communication messaging in conjunction with transportation e-voucher led to an increased uptake of cervical cancer screening (Table 2) [38].

#### Findings From the Review of Secondary Objectives: Description of Strategies Used

We adapted a published mHealth framework for noncommunicable diseases to facilitate the narrative synthesis of the digital health strategies used in the included studies [4,60]. Framework adaptation allowed for the mapping of included studies to various stages of the cervical cancer control cascade (primary prevention, secondary prevention, treatment, and palliation) as well as the key individual, provider, and health system challenges (knowledge, access, quality, and continuity of care) addressed by the included studies. The mapping results are presented in Table 3. Only the digital health components of the study interventions were mapped and any nondigital components (community sensitization, paper educational booklets administered before digital reminders, etc) were excluded. Many studies addressed multiple challenges; hence, digital health strategies were mapped to all the applicable challenges in the framework.

**Table 3:** Landscape of digital health strategies for cervical cancer prevention and control.

Individual-, provider- and health system–level challenges in cervical cancer control			Stages in cervical cancer prevention and control								
			Primary prevention		Secondary prevention				Treatment and palliative care		
			HPV^a^ vaccination		Screening (study)		Treatment of precancerous lesions (study)		Treatment		Palliative care
**Knowledge and awareness**											
	Low knowledge of HPV or cervical cancer	Not within the scope of this review		5 [34,38,40,58,59]		1 [34]		0		0	
	Low knowledge of cervical cancer screening or treatment services	Not within the scope of this review		6 [34,38,40,41,58,59]^b^		1 [34]		0		0	
**Access to care**											
	Low access to health facilities or cervical cancer services	Not within the scope of this review		1 [38]		0		0		0	
	Low availability of appropriate and accurate screening or treatment methods	Not within the scope of this review		15 [30,31,35,36,42-46, 50,51,53-57]^c^		0		0		N/A	
	Low access to experts	Not within the scope of this review		15 [30,31,35,36,42-46, 50,51,53-57]		0		0		0	
	Financial barriers	Not within the scope of this review		1 [38]		0		0		0	
**Continuity of care**											
	A low uptake of follow-up services	Not within the scope of this review		7 [33,39,41,47-49, 52,58,59]		0		0		N/A	
**Quality of care**											
	A lack of training opportunities for health workers	Not within the scope of this review		10 [30,31,35,36,42-44, 46,51,56,57]^c^		0		0		0	
	Poor data availability	Not within the scope of this review		19 [30-32,35-37,39, 42-46,50-57]		2 [32,45]		0		0	

^a^HPV: human papillomavirus.

^b^Represents the number of included studies mapped to each category. For example, we found 3 studies that aimed to increase the demand for screening by increasing women’s knowledge of human papillomavirus or cervical cancer.

^c^Some studies were associated with multiple records, for example, Asgary et al [30,31].

A majority of the included studies used digital health strategies for secondary prevention; only Caster et al [34] focused on educating women about treatment, whereas Bhatt et al [32] and Peterson et al [45] collected data related to postscreening treatment. None of the included studies focused on cervical cancer treatment or palliative care. Studies related to primary prevention (HPV vaccination) were not within the scope of the study. Although most included studies focused on increasing screening or treatment among all eligible women, some studies focused on high-risk populations, and in particular, HPV-positive women [35-37,50,53,54,59].

1. *Knowledge and awareness*: in total, 6 studies mapped to this domain and included digital health strategies to educate women about HPV, cervical cancer, and cervical cancer prevention or treatment services. These studies [34,38,40,41,58,59] focused primarily on increasing the screening uptake: Khademolhosseini et al [40] used an educational intervention delivered via an instant messaging platform called Telegram. They used a diverse range of content including text messages, posters, infographics, podcasts, and video tutorials. Lima et al [41] tested a telephone intervention focused on increasing the patient’s knowledge of cervical cancer and Pap smears. In the study by Erwin et al [38], women in one study arm received 15 behavior change communication messages via SMS messages that were designed to increase their knowledge and awareness about cervical cancer screening. Caster et al [34] implemented a tablet-based cervical cancer education program to facilitate patient education about screening. Opportunities for interaction with the educational content were through quizzes, or while navigating the content on the tablet. Caster et al [34] also included educational content related to treatment and was the only study to do so.
2. *Access to care*: only one study, by Erwin et al [38], used digital health to reduce transportation and financial barriers to health care access. Women randomized to one of the study arms received e-vouchers through their mobile phone, which covered the costs of a return trip to the health facility. In total, 15 studies used smartphones for facilitating digital cervicography and visualizing the cervix during VIA at the point of care [30,31,35,36,42-46,50,51,53-57]. In total, 7 studies included visual inspection with Lugol iodine images in addition to VIA images [35-37,50,53-55]. Although most studies used the mobile phone camera for image acquisition, Peterson et al [45] and Taghavi et al [53] used smartphone attachments for enhanced cervix visualization. These studies allowed for task shifting, as mobile devices were used to acquire cervical images, record diagnosis, and receive or compare diagnoses with remote experts. Images and relevant patient data were shared with the experts via a text/multimedia message service or by uploading data to a web-based database. Parham et al [44] used an automated text messaging system to reduce the time between screening, diagnosis, and treatment by sending a text message requesting expert review of an image while the patient was still in the clinic. Yeates et al [56,57] used WhatsApp to send patient images, nurse diagnosis, and treatment plans for expert review and subsequently developed the SEVIA app to accomplish these tasks.
3. *Continuity of care*: in total, 7 studies tested digital strategies for reminding or recalling patients for follow-up services [33,39,41,47-49,52,58,59]. These studies focused on women who had already received some cervical cancer services. All 7 studies tested SMS-based reminders or recall messages. In total, 3 studies [39,47-49,52] also tested phone calls. Other nondigital modalities included letters, registered letters, home visits, and return visits to the clinic.
4. *Quality of care*: studies mapped to this domain focused on 2 key applications: training health workers and improving data availability. In most cases, the training covered the acquisition of good-quality images using cervicography and expert feedback to improve the accuracy of diagnosis [30,31,35,36,42-44,46,51,56,57]. In Botswana, Littman-Quinn et al [42] provided additional ongoing medical education content to health workers via tablet devices to complement in-service training. Studies using digital cervicography were also mapped to this domain as the documentation of cervical images during VIA can improve data availability for patient management, and hence, improve the quality of care. Two studies, Bhatt et al [32] and Peterson et al [45], collected treatment information in addition to information about screening results. In the study by Bhatt et al [32], a SIM-based app was loaded on feature phones provided to trained nurses. The nurses used a menu-based protocol on the feature phone for entering data.

#### Findings From the Review of Secondary Objectives: Implementation Challenges

In the included studies, the authors described several technical challenges in implementing digital health strategies for cervical cancer control in LMICs. These challenges were based on *lessons learned* and, in a few cases [31,32], on formal qualitative data collected during the study. In addition to the technical challenges, many studies described inadequacies in the underlying health system resources (eg, availability of pelvic exam rooms and lack of supplies for cryotherapy in community health centers [30]), which impacted study implementation. Digital health strategies were not implemented in a vacuum; several studies described the need for increasing the community knowledge and awareness of cervical cancer as well as reducing stigma and fear related to cancer screening/diagnosis in parallel with the implementation of digital health strategies [30,32,37,38,41,44,45,51]. One study also described negative attitudes toward cervical cancer screening among health care workers as an issue [51]. Some authors emphasized the need for strong partnerships and stakeholder support for the success and future sustainability of digital health strategies [42,43,45,51]. Table 4 shows the technical challenges in implementing digital health strategies for cervical cancer control.

**Table 4:** Description of implementation challenges for digital health strategies for cervical cancer control.

Implementation challenge	Included studies	Description and examples
High training requirements	[30,31,34,35,43,44,51,54]	Pretraining lasting several weeks, providing supplemental training for augmenting skills, refresher training to minimize loss of skills, and the availability of experts to provide ongoing feedback for using digital cervicography Catarino et al [35]: 5 weeks of training on digital cervicography to medical students Asgary et al [30,31]: additional one-on-one training to community health nurses in cases where the digital image quality was low Ndlovu et al [43]: high training requirements on the touchscreen features Caster et al [34]: Users had limited previous experience with technology (but needed very little support for using the tablet device).
Technology-specific challenges	[30,32,36,42-44,46,50,54,55]	Procurement of appropriate technology Challenges with the availability of technology options in the study area in preparation for and during the study [30,32] Considerations related to finding mobile phone cameras with high image quality and zoom capabilities [30,43,46,54,55] The use of high-pixel smartphone cameras were associated with better reported quality of images [50]; however, Tran et al [54] suggested that the quality was inferior to colposcopy images. Parham et al [44]: need to send cameras out of the country for repairs was a challenge Software and hardware issues: software “bugs,” crashing of apps, device malfunctions, and an insufficient battery life [36,42-44] Gallay et al [36]: loss of patient data due to the unexpected crash of their data collection app Data security issues Littman-Quinn et al [42]: security breach (attack by an anonymous hacker) lead researchers to increase data security to the level of compliance described in the Health Insurance Portability and Accountability Act Software updates Bhatt et al [32]: challenges in updating a SIM-based app requiring project staff to collect all SIM cards to update the app Littman-Quinn et al [42]: challenges with communication when the technical team was based in a different country and did not speak the same language as the end users, reflecting the need for local technology development capacity for the sustainability of digital health strategies
Infrastructure challenges	[31,32,42,43,45,46]	Issues with network coverage and electrical outages as limitations to widespread implementation Bhatt et al [32]: challenges faced by nurses from hillier communities in sending patient data and receiving acknowledgment of report submission Yeates et al [56]: health care workers given solar-powered chargers and light sources for anticipated power outages and to allow use of digital cervicography in off-site settings
Challenges with technology reach	[39,47-49]	Rashid et al [47-49]: letters were more likely to be successfully and reliably delivered to patients than phone text messages or calls because of connectivity and coverage issues (incorrect phone numbers and nonresponse) Rashid et al [47-49] and Huchko et al [39]: direct communication through phone call encouraged more women to seek screening (both studies had a high risk of bias, limiting our confidence in their findings)
Inequitable access to technology	[38,39,52]	Exclusion of women without mobile phones from digital health intervention components in some studies

### Additional Analysis: Quality of Digital Health Reporting

The quality of reporting scores according to the mERA checklist is summarized in Multimedia Appendix 5 [30-59]. The mERA scores ranged from 1 to 10, out of a maximum possible score of 16. The mERA checklist items frequently described in the studies included the technology platform and details of intervention delivery. Poorly described items were related to digital health infrastructure necessary for implementation, interoperability with other existing digital health systems, usability testing during development, and intervention replicability, with 6 or fewer included studies describing these characteristics.

## Discussion

### Summary of Evidence

Our review findings show that the majority of the studies in LMICs used digital health strategies to facilitate the screening and treatment of precancerous lesions (ie, secondary prevention) as compared with the treatment of invasive cancer or palliative care. Even though our search attempted to include any relevant studies since 1992, the date of the earliest included study was 2010. Within the realm of secondary prevention, strategies focused on improving women’s knowledge and awareness about cervical cancer, increasing access to cervical cancer services, improving the training of health workers and availability of data, and ensuring the continuity of care. Key challenges in implementing digital health strategies for cervical cancer control were related to the high burden of training, technology-specific issues, infrastructure challenges, challenges with technology reach, and inequitable access to technology among target users. We were unable to determine the quantitative effect of digital health strategies on cervical cancer control because of the small numbers and inadequate quality of studies for meta-analysis. Only one randomized controlled study identified in this review with a low risk of bias found that SMS behavior change communication messaging in conjunction with a transportation e-voucher leads to an increased uptake of cervical cancer screening. Most of the studies included in the review had a high risk of bias and were rated poorly in terms of the quality of reporting of the digital health strategy.

### Implications for Research and Practice

This review identified several gaps in the literature. These gaps are summarized below, along with their implications for research and practice:

1. Improve the evidence base for the effectiveness of digital health strategies for cervical cancer control: there is insufficient evidence related to the effectiveness of digital health strategies for cervical cancer screening and treatment. The included studies implemented digital health strategies mostly for secondary prevention, and there are opportunities to investigate the use of digital health for cervical cancer treatment and palliative care. Other bottlenecks in the cervical cancer control cascade that may benefit from using digital health include improving access to health facilities (eg, through the use of digital telemedicine), reducing financial barriers (eg, provision of phone vouchers), and supporting disease management among women diagnosed with invasive cancer (eg, using digital knowledge interventions).
2. Use more rigorous study designs: among the 27 included studies, only 4 studies used an RCT design [38,40,47-49,59] and only one of these studies had a low risk of bias [38]. The high or moderate risk of bias among the remaining studies limited our confidence in their findings. Future studies should consider using rigorous study designs that minimize the risk of bias.
3. Improve the reporting of digital health strategies in the literature: in our review, only 5 studies [35-37,39,54,57,59] met the mERA checklist item on replicability, indicating that many of the digital health strategies identified in this review would be difficult to replicate and re-evaluate based on published information. The use of reporting checklists ensures that all relevant information is presented to readers to assist with study reproducibility.
4. Expand research on LMIC settings in Asia and South America: a majority (70%) of the included studies took place in sub-Saharan Africa, indicating an opportunity to expand research to other LMIC settings with a high burden of cervical cancer.

### Limitations

Our synthesis of the literature is limited by the availability of peer-reviewed reports of digital health strategies for cervical cancer control. We tried to mitigate this limitation by using a systematic search strategy and searching 5 large databases and Google to identify relevant studies. We did not search any trial databases for ongoing studies or to examine other gray literature sources. Hence, we may have missed ongoing implementations and emerging data on using digital health for cervical cancer control.

### Conclusions

There is insufficient evidence to determine the effectiveness of digital health strategies for cervical cancer control in LMICs. The only RCT study identified in this review with a low risk of bias found that SMS behavior change communication messaging in conjunction with a transportation e-voucher led to an increased uptake of cervical cancer screening. Future efforts are needed to investigate the use of digital health strategies across the cervical cancer control continuum and in LMIC settings outside of sub-Saharan Africa.

## Appendix

Multimedia Appendix 1 Protocol deviations and unused methods.

Multimedia Appendix 2 PRISMA (Preferred Reporting Items for Systematic Reviews and Meta-Analyses) checklist.

Multimedia Appendix 3 Systematic search strategy, customized by database.

Multimedia Appendix 4 Characteristics of included studies.

Multimedia Appendix 5 Quality of reporting of including studies using the mHealth evidence reporting and assessment (mERA) checklist.

## Abbreviations

HPV: human papillomavirus
LMIC: low- and middle-income country
mERA: mobile health evidence reporting and assessment
mHealth: mobile health
PRISMA: Preferred Reporting Items for Systematic Reviews and Meta-Analyses
RCT: randomized controlled trial
VIA: visual inspection with acetic acid
