# Supplementary material for: Digital Health Strategies for Cervical Cancer Control in Low- and Middle-Income Countries: Systematic Review of Current Implementations and Gaps in Research
Source: J Med Internet Res. 2021 May 27;23(5):e23350. doi: 10.2196/23350 (PMC8193495; doi:10.2196/23350)
Supplement: Multimedia Appendix 1 [file jmir_v23i5e23350_app1.docx]

**Multimedia Appendix 1: Protocol deviations and unused methods**

**1. Deviations from the protocol**

- The Prospero protocol indicated that seven databases were to be searched. In the initial search, the PsychINFO and ProQuest searches did not yield any relevant sources, and were thus not included in final searches.
- Studies included in the review of secondary objectives were mapped to a previously-described mHealth framework for non-communicable diseases.

2. Unused methods

- **Risk of bias across studies:** As meta-analyses were not possible, we did not assess the risk of bias across studies.
