# Supplementary material for: Digital Health Strategies for Cervical Cancer Control in Low- and Middle-Income Countries: Systematic Review of Current Implementations and Gaps in Research
Source: J Med Internet Res. 2021 May 27;23(5):e23350. doi: 10.2196/23350 (PMC8193495; doi:10.2196/23350)
Supplement: Multimedia Appendix 3 [file jmir_v23i5e23350_app3.docx]

**Multimedia Appendix 3: Systematic search strategy, customized by database**

**3A: PUBMED**

| **Set #** | **Search terms** |
| --- | --- |
| 1 | "Mobile Applications"[Mesh] OR "Cell Phone"[Mesh] OR "Text Messaging"[Mesh] OR "Computers, Handheld"[Mesh] OR "Telemedicine"[Mesh] OR cellphone[tiab] OR cellphones[tiab] OR mobile[tiab] OR mobiles[tiab] OR smartphone[tiab] OR smartphones[tiab] OR telephone[tiab] OR telephones[tiab] OR phone[tiab] OR phones[tiab] OR "e-health"[tiab] OR ehealth[tiab] OR "m-health"[tiab] OR mhealth[tiab] OR "automated telephone"[tiab] OR IVR[tiab] OR "interactive voice response"[tiab] OR "voice message"[tiab] OR "voice messages"[tiab] OR voicemail[tiab] OR "voice mail"[tiab] OR sms[tiab] OR text[tiab] OR texts[tiab] OR mms[tiab] OR blackberry[tiab] OR pda[tiab] OR pdas[tiab] OR ipad[tiab] OR ipads[tiab] OR android[tiab] OR laptop[tiab] OR laptops[tiab] OR ((tablet[tiab] OR tablets[tiab]) AND (computer[tiab] OR computers[tiab] OR device[tiab] OR devices[tiab])) |
| 2 | “Papillomavirus Infections"[mesh] OR "Uterine Cervical Neoplasms"[Mesh] OR "Cervical Intraepithelial Neoplasia"[mesh] OR "Vaginal Smears"[mesh] OR "Carcinoma, Squamous Cell"[Mesh] OR "Cervix Uteri"[Mesh] OR "Papanicolaou Test"[Mesh] OR "Papillomaviridae"[Mesh] OR "Human Papillomavirus DNA Tests"[Mesh] OR "Vagina"[Mesh] OR cervix[tiab] OR cervices[tiab] OR cervical[tiab] OR colposcopy[tiab] OR colposcopies[tiab] OR colposcope[tiab] OR colposcopes[tiab] OR cervicography[tiab] OR "pap test"[tiab] OR "pap tests"[tiab] OR "pap smear"[tiab] OR "pap smears"[tiab] OR "visual inspection with acetic acid"[tiab] OR papillomavirus[tiab] OR HPV[tiab] |
| 3 | "Developing Countries"[Mesh] OR "Afghanistan"[Mesh] OR "Bangladesh"[Mesh] OR "Benin"[Mesh] OR "Burkina Faso"[Mesh] OR "Burundi"[Mesh] OR "Cambodia"[Mesh] OR "Central African Republic"[Mesh] OR "Chad"[Mesh] OR "Comoros"[Mesh] OR "Democratic Republic of the Congo"[Mesh] OR "Eritrea"[Mesh] OR "Ethiopia"[Mesh] OR "Gambia"[Mesh] OR "Guinea"[Mesh] OR "Guinea-Bissau"[Mesh] OR "Haiti"[Mesh] OR "Kenya"[Mesh] OR "Democratic People's Republic of Korea"[Mesh] OR "Liberia"[Mesh] OR "Madagascar"[Mesh] OR "Malawi"[Mesh] OR "Mali"[Mesh] OR "Mozambique"[Mesh] OR "Myanmar"[Mesh] OR "Nepal"[Mesh] OR "Niger"[Mesh] OR "Rwanda"[Mesh] OR "Sierra Leone"[Mesh] OR "Somalia"[Mesh] OR "Tajikistan"[Mesh] OR "Tanzania"[Mesh] OR "Togo"[Mesh] OR "Uganda"[Mesh] OR "Zimbabwe"[Mesh] OR "Armenia"[Mesh] OR "Bhutan"[Mesh] OR "Bolivia"[Mesh] OR "Cameroon"[Mesh] OR "Cabo Verde"[Mesh] OR "Congo"[Mesh] OR "Cote d'Ivoire"[Mesh] OR "Djibouti"[Mesh] OR "Egypt"[Mesh] OR "El Salvador"[Mesh] OR "Georgia (Republic)"[Mesh] OR "Ghana"[Mesh] OR "Guatemala"[Mesh] OR "Guyana"[Mesh] OR "Honduras"[Mesh] OR "Indonesia"[Mesh] OR "India"[Mesh] OR "Kosovo"[Mesh] OR "Kyrgyzstan"[Mesh] OR "Laos"[Mesh] OR "Lesotho"[Mesh] OR "Mauritania"[Mesh] OR "Micronesia"[Mesh] OR "Moldova"[Mesh] OR "Mongolia"[Mesh] OR "Morocco"[Mesh] OR "Nicaragua"[Mesh] OR "Nigeria"[Mesh] OR "Pakistan"[Mesh] OR "Papua New Guinea"[Mesh] OR "Paraguay"[Mesh] OR "Philippines"[Mesh] OR "Independent State of Samoa"[Mesh] OR "Atlantic Islands"[Mesh] OR "Senegal"[Mesh] OR "Melanesia"[Mesh] OR "Sri Lanka"[Mesh] OR "Sudan"[Mesh] OR "Swaziland"[Mesh] OR "Syria"[Mesh] OR "Timor-Leste"[Mesh] OR "Ukraine"[Mesh] OR "Uzbekistan"[Mesh] OR "Vanuatu"[Mesh] OR "Vietnam"[Mesh] OR "Middle East"[Mesh] OR "Yemen"[Mesh] OR "Zambia"[Mesh] OR "Angola"[Mesh] OR "Albania"[Mesh] OR "Algeria"[Mesh] OR "American Samoa"[Mesh] OR "Argentina"[Mesh] OR "Azerbaijan"[Mesh] OR "Republic of Belarus"[Mesh] OR "Belize"[Mesh] OR "Bosnia and Herzegovina"[Mesh] OR "Botswana"[Mesh] OR "Brazil"[Mesh] OR "Bulgaria"[Mesh] OR "China"[Mesh] OR "Colombia"[Mesh] OR "Costa Rica"[Mesh] OR "Cuba"[Mesh] OR "Dominica"[Mesh] OR "Dominican Republic"[Mesh] OR "Ecuador"[Mesh] OR "Equatorial Guinea"[Mesh] OR "Fiji"[Mesh] OR "Gabon"[Mesh] OR "Grenada"[Mesh] OR "Iran"[Mesh] OR "Iraq"[Mesh] OR "Jamaica"[Mesh] OR "Jordan"[Mesh] OR "Kazakhstan"[Mesh] OR "Lebanon"[Mesh] OR "Libya"[Mesh] OR "Macedonia (Republic)"[Mesh] OR "Malaysia"[Mesh] OR "Indian Ocean Islands"[Mesh] OR "Mexico"[Mesh] OR "Montenegro"[Mesh] OR "Namibia"[Mesh] OR "Palau"[Mesh] OR "Panama"[Mesh] OR "Peru"[Mesh] OR "Romania"[Mesh] OR "Russia"[Mesh] OR "Serbia"[Mesh] OR "Seychelles"[Mesh] OR "South Africa"[Mesh] OR "Saint Lucia"[Mesh] OR "Saint Vincent and the Grenadines"[Mesh] OR "Suriname"[Mesh] OR "Thailand"[Mesh] OR "Tonga"[Mesh] OR "Tunisia"[Mesh] OR "Turkey"[Mesh] OR "Turkmenistan"[Mesh] OR "Venezuela"[Mesh] OR "Afghanistan"[all fields] OR "Bangladesh"[all fields] OR "Benin"[all fields] OR "Burkina Faso"[all fields] OR "Burundi"[all fields] OR "Cambodia"[all fields] OR "cabo verde"[all fields] OR "Central African Republic"[all fields] OR "Chad"[all fields] OR "Comoros"[all fields] OR "Democratic Republic of the Congo"[all fields] OR "Eritrea"[all fields] OR "Ethiopia"[all fields] OR "Gambia"[all fields] OR "Guinea"[all fields] OR "Guinea-Bissau"[all fields] OR "Haiti"[all fields] OR "Kenya"[all fields] OR "Democratic People's Republic of Korea"[all fields] OR "Liberia"[all fields] OR "Madagascar"[all fields] OR "Malawi"[all fields] OR "Mali"[all fields] OR "Mozambique"[all fields] OR "Myanmar"[all fields] OR "Nepal"[all fields] OR "Niger"[all fields] OR "Rwanda"[all fields] OR "Sierra Leone"[all fields] OR "Somalia"[all fields] OR "Tajikistan"[all fields] OR "Tanzania"[all fields] OR "Togo"[all fields] OR "Uganda"[all fields] OR "Zimbabwe"[all fields] OR "Armenia"[all fields] OR "Bhutan"[all fields] OR "Bolivia"[all fields] OR "Cameroon"[all fields] OR "Cape Verde"[all fields] OR "Congo"[all fields] OR "Cote d'Ivoire"[all fields] OR "Djibouti"[all fields] OR "Egypt"[all fields] OR "El Salvador"[all fields] OR "Georgia (Republic)"[all fields] OR "Ghana"[all fields] OR "Guatemala"[all fields] OR "Guyana"[all fields] OR "Honduras"[all fields] OR "Indonesia"[all fields] OR "India"[all fields] OR “Kiribati”[all fields] OR "Kosovo"[all fields] OR "Kyrgyzstan"[all fields] OR "Kyrgyz"[all fields] OR "Laos"[all fields] OR "lao"[all fields] OR "Lesotho"[all fields] OR "Mauritania"[all fields] OR "Micronesia"[all fields] OR "Moldova"[all fields] OR "Mongolia"[all fields] OR "Morocco"[all fields] OR "Nicaragua"[all fields] OR "Nigeria"[all fields] OR "Pakistan"[all fields] OR "Papua New Guinea"[all fields] OR "Paraguay"[all fields] OR "Philippines"[all fields] OR "Independent State of Samoa"[all fields] OR "Atlantic Islands"[all fields] OR "Sao Tome"[all fields] OR Principe[all fields] OR "Senegal"[all fields] OR "Melanesia"[all fields] OR "Solomon islands"[all fields] OR "Sri Lanka"[all fields] OR "Sudan"[all fields] OR "Swaziland"[all fields] OR "Syria"[all fields] OR "East Timor"[all fields] OR "Timor leste"[all fields] OR "Ukraine"[all fields] OR "Uzbekistan"[all fields] OR "Vanuatu"[all fields] OR "Vietnam"[all fields] OR "Middle East"[all fields] OR "west bank"[all fields] OR "Gaza"[all fields] OR "Yemen"[all fields] OR "Zambia"[all fields] OR "Angola"[all fields] OR "Albania"[all fields] OR "Algeria"[all fields] OR "Argentina"[all fields] OR "Samoa"[all fields] OR "Azerbaijan"[all fields] OR "Republic of Belarus"[all fields] OR "Belize"[all fields] OR "Bosnia-Herzegovina"[all fields] OR "Botswana"[all fields] OR "Brazil"[all fields] OR "Bulgaria"[all fields] OR "China"[all fields] OR "Colombia"[all fields] OR "Costa Rica"[all fields] OR "Cuba"[all fields] OR "Dominica"[all fields] OR "Dominican Republic"[all fields] OR "Ecuador"[all fields] OR "Equatorial Guinea"[all fields] OR "Fiji"[all fields] OR "Gabon"[all fields] OR "Grenada"[all fields] OR "Iran"[all fields] OR "Iraq"[all fields] OR "Jamaica"[all fields] OR "Jordan"[all fields] OR "Kazakhstan"[all fields] OR "Lebanon"[all fields] OR "Libya"[all fields] OR "Macedonia"[all fields] OR "Malaysia"[all fields] OR "Indian Ocean Islands"[all fields] OR "Maldives"[all fields] OR “Marshall Islands”[all fields] OR "Mauritius"[all fields] OR "Mexico"[all fields] OR "Montenegro"[all fields] OR "Namibia"[all fields] OR "Palau"[all fields] OR "Panama"[all fields] OR "Peru"[all fields] OR "Romania"[all fields] OR "Russia"[all fields] OR "Russian Federation"[all fields] OR "Serbia"[all fields] OR "Seychelles"[all fields] OR "South Africa"[all fields] OR "Saint Lucia"[all fields] OR "Saint Vincent and the Grenadines"[all fields] OR "Suriname"[all fields] OR "Thailand"[all fields] OR "Tonga"[all fields] OR "Tunisia"[all fields] OR "Turkey"[all fields] OR "Turkmenistan"[all fields] OR "Tuvalu"[all fields] OR "Venezuela"[all fields] OR "low resource"[all fields] OR "under-resourced"[all fields] OR "resource poor"[all fields] OR "under-developed"[all fields] OR "underdeveloped"[all fields] OR "developing country"[all fields] OR "developing countries"[all fields] OR "developing world"[all fields] OR "third world" [all fields] OR lmic[all fields] OR (low[all fields] AND middle[all fields] AND income[all fields]) |
| 4 | #1 AND #2 AND #3 |
| 5 | #4, published 1992-present |

**3B. EMBASE**

| **Set #** | **Search terms** |
| --- | --- |
| 1 | 'mobile application'/exp OR 'mobile phone'/exp OR 'personal digital assistant'/exp OR 'telemedicine'/exp OR 'text messaging'/exp OR cellphone:ab,ti OR cellphones:ab,ti OR mobile:ab,ti OR mobiles:ab,ti OR smartphone:ab,ti OR smartphones:ab,ti OR telephone:ab,ti OR telephones:ab,ti OR phone:ab,ti OR phones:ab,ti OR "e-health":ab,ti OR ehealth:ab,ti OR "m-health":ab,ti OR mhealth:ab,ti OR "automated telephone":ab,ti OR IVR:ab,ti OR "interactive voice response":ab,ti OR "voice message":ab,ti OR "voice messages":ab,ti OR voicemail:ab,ti OR "voice mail":ab,ti OR sms:ab,ti OR text:ab,ti OR texts:ab,ti OR mms:ab,ti OR blackberry:ab,ti OR pda:ab,ti OR pdas:ab,ti OR ipad:ab,ti OR ipads:ab,ti OR android:ab,ti OR laptop:ab,ti OR laptops:ab,ti OR ((tablet:ab,ti OR tablets:ab,ti) AND (computer:ab,ti OR computers:ab,ti OR device:ab,ti OR devices:ab,ti)) |
| 2 | 'Papillomaviridae'/exp OR 'papillomavirus infection'/exp OR 'uterine cervix tumor'/exp OR 'uterine cervix carcinoma in situ'/exp OR 'vagina smear'/exp OR 'squamous cell carcinoma'/exp OR 'Papanicolaou test'/exp OR 'Human papillomavirus DNA test'/exp OR 'vagina'/exp OR 'uterine cervix'/exp OR ((cervix:ab,ti OR cervices:ab,ti OR cervical:ab,ti) AND (cancer*:ab,ti OR carcinoma*:ab,ti OR neoplas*:ab,ti OR uter*:ab,ti OR screen*:ab,ti)) OR colposcopy:ab,ti OR colposcopies:ab,ti OR colposcope:ab,ti OR colposcopes:ab,ti OR cervicography:ab,ti OR "pap test":ab,ti OR "pap tests":ab,ti OR "pap smear":ab,ti OR "pap smears":ab,ti OR "visual inspection with acetic acid":ab,ti OR papillomavirus:ab,ti OR HPV:ab,ti |
| 3 | 'developing country'/exp OR 'Afghanistan'/exp OR 'Bangladesh'/exp OR 'Benin'/exp OR 'Burkina Faso'/exp OR 'Burundi'/exp OR 'Cambodia'/exp OR 'Central African Republic'/exp OR 'Chad'/exp OR 'Comoros'/exp OR 'Democratic Republic Congo'/exp OR 'Congo'/exp OR 'Eritrea'/exp OR 'Ethiopia'/exp OR 'Gambia'/exp OR 'Guinea'/exp OR 'Guinea-Bissau'/exp OR 'Haiti'/exp OR 'Kenya'/exp OR 'North Korea'/exp OR 'Liberia'/exp OR 'Madagascar'/exp OR 'Malawi'/exp OR 'Mozambique'/exp OR 'Myanmar'/exp OR 'Nepal'/exp OR 'Niger'/exp OR 'Nigeria'/exp OR 'Rwanda'/exp OR 'Sierra Leone'/exp OR 'Somalia'/exp OR 'Tajikistan'/exp OR 'Tanzania'/exp OR 'Togo'/exp OR 'Uganda'/exp OR 'Zimbabwe'/exp OR 'Armenia'/exp OR 'Bhutan'/exp OR 'Bolivia'/exp OR 'Cameroon'/exp OR 'Cape Verde'/exp OR 'Cote d`Ivoire'/exp OR 'Djibouti'/exp OR 'Egypt'/exp OR 'El Salvador'/exp OR 'Georgia (republic)'/exp OR 'Ghana'/exp OR 'Guatemala'/exp OR 'Guyana'/exp OR 'Honduras'/exp OR 'Indonesia'/exp OR 'India'/exp OR 'Kosovo'/exp OR 'Kyrgyzstan'/exp OR 'Laos'/exp OR 'Lesotho'/exp OR 'Mauritania'/exp OR 'Federated States of Micronesia'/exp OR 'Moldova'/exp OR 'Mongolia'/exp OR 'Nicaragua'/exp OR 'Pakistan'/exp OR 'Papua New Guinea'/exp OR 'Philippines'/exp OR 'Samoa'/exp OR 'Sao Tome and Principe'/exp OR 'Senegal'/exp OR 'Solomon Islands'/exp OR 'Sri Lanka'/exp OR 'Sudan'/exp OR 'Swaziland'/exp OR 'Syrian Arab Republic'/exp OR 'Timor-Leste'/exp OR 'Ukraine'/exp OR 'Uzbekistan'/exp OR 'Vanuatu'/exp OR 'Viet Nam'/exp OR 'Yemen'/exp OR 'Zambia'/exp OR 'Angola'/exp OR 'Albania'/exp OR 'Algeria'/exp OR 'American Samoa'/exp OR 'Argentina'/exp OR 'Azerbaijan'/exp OR 'Belarus'/exp OR 'Belize'/exp OR 'Bosnia and Herzegovina'/exp OR 'Botswana'/exp OR 'Brazil'/exp OR 'Bulgaria'/exp OR 'China'/exp OR 'Colombia'/exp OR 'Costa Rica'/exp OR 'Cuba'/exp OR 'Dominica'/exp OR 'Dominican Republic'/exp OR 'Ecuador'/exp OR 'Equatorial Guinea'/exp OR 'Fiji'/exp OR 'Gabon'/exp OR 'Grenada'/exp OR 'Iran'/exp OR 'Iraq'/exp OR 'Jamaica'/exp OR 'Jordan'/exp OR 'Kazakhstan'/exp OR 'Lebanon'/exp OR 'Libyan Arab Jamahiriya'/exp OR 'Macedonia (republic)'/exp OR 'Malaysia'/exp OR 'Maldives'/exp OR 'Mexico'/exp OR 'Montenegro (republic)'/exp OR 'Namibia'/exp OR 'Palau'/exp OR 'Panama'/exp OR 'Peru'/exp OR 'Romania'/exp OR 'Russian Federation'/exp OR 'Serbia'/exp OR 'Seychelles'/exp OR 'South Africa'/exp OR 'Saint Lucia'/exp OR 'Saint Vincent and the Grenadines'/exp OR 'Suriname'/exp OR 'Thailand'/exp OR 'Tonga'/exp OR 'Tunisia'/exp OR 'Turkey (republic)'/exp OR 'Turkmenistan'/exp OR 'Venezuela'/exp OR 'Afghanistan':ab,ti,ca OR 'Bangladesh':ab,ti,ca OR 'Benin':ab,ti,ca OR 'Burkina Faso':ab,ti,ca OR 'Burundi':ab,ti,ca OR 'Cambodia':ab,ti,ca OR 'cabo verde':ab,ti,ca OR 'Central African Republic':ab,ti,ca OR 'Chad':ab,ti,ca OR 'Comoros':ab,ti,ca OR 'Congo':ab,ti,ca OR 'Eritrea':ab,ti,ca OR 'Ethiopia':ab,ti,ca OR 'Gambia':ab,ti,ca OR 'Guinea':ab,ti,ca OR 'Haiti':ab,ti,ca OR 'Kenya':ab,ti,ca OR 'Korea':ab,ti,ca OR 'Liberia':ab,ti,ca OR 'Madagascar':ab,ti,ca OR 'Malawi':ab,ti,ca OR 'Mali':ab,ti,ca OR 'Mozambique':ab,ti,ca OR 'Myanmar':ab,ti,ca OR 'Nepal':ab,ti,ca OR 'Niger':ab,ti,ca OR 'Rwanda':ab,ti,ca OR 'Sierra Leone':ab,ti,ca OR 'Somalia':ab,ti,ca OR 'Tajikistan':ab,ti,ca OR 'Tanzania':ab,ti,ca OR 'Togo':ab,ti,ca OR 'Uganda':ab,ti,ca OR 'Zimbabwe':ab,ti,ca OR 'Armenia':ab,ti,ca OR 'Bhutan':ab,ti,ca OR 'Bolivia':ab,ti,ca OR 'Cameroon':ab,ti,ca OR 'Cape Verde':ab,ti,ca OR 'Congo':ab,ti,ca OR 'Cote dIvoire':ab,ti,ca OR 'ivory coast':ab,ti,ca OR 'Djibouti':ab,ti,ca OR 'Egypt':ab,ti,ca OR 'El Salvador':ab,ti,ca OR 'Georgia':ab,ti,ca OR 'Ghana':ab,ti,ca OR 'Guatemala':ab,ti,ca OR 'Guyana':ab,ti,ca OR 'Honduras':ab,ti,ca OR 'Indonesia':ab,ti,ca OR 'India':ab,ti,ca OR 'Kiribati':ab,ti,ca OR 'Kosovo':ab,ti,ca OR 'Kyrgyzstan':ab,ti,ca OR 'Kyrgyz':ab,ti,ca OR 'Laos':ab,ti,ca OR 'lao':ab,ti,ca OR 'Lesotho':ab,ti,ca OR 'Mauritania':ab,ti,ca OR 'Micronesia':ab,ti,ca OR 'Moldova':ab,ti,ca OR 'Mongolia':ab,ti,ca OR 'Morocco':ab,ti,ca OR 'Nicaragua':ab,ti,ca OR 'Nigeria':ab,ti,ca OR 'Pakistan':ab,ti,ca OR 'Papua New Guinea':ab,ti,ca OR 'Paraguay':ab,ti,ca OR 'Philippines':ab,ti,ca OR 'Samoa':ab,ti,ca OR 'Atlantic Islands':ab,ti,ca OR 'Sao Tome':ab,ti,ca OR Principe:ab,ti,ca OR 'Senegal':ab,ti,ca OR 'Melanesia':ab,ti,ca OR 'Solomon islands':ab,ti,ca OR 'Sri Lanka':ab,ti,ca OR 'Sudan':ab,ti,ca OR 'Swaziland':ab,ti,ca OR 'Syria':ab,ti,ca OR 'East Timor':ab,ti,ca OR 'Timor leste':ab,ti,ca OR 'Ukraine':ab,ti,ca OR 'Uzbekistan':ab,ti,ca OR 'Vanuatu':ab,ti,ca OR 'Vietnam':ab,ti,ca OR 'Middle East':ab,ti,ca OR 'west bank':ab,ti,ca OR 'Gaza':ab,ti,ca OR 'Yemen':ab,ti,ca OR 'Zambia':ab,ti,ca OR 'Angola':ab,ti,ca OR 'Albania':ab,ti,ca OR 'Algeria':ab,ti,ca OR 'Argentina':ab,ti,ca OR 'Samoa':ab,ti,ca OR 'Azerbaijan':ab,ti,ca OR 'Republic of Belarus':ab,ti,ca OR 'Belize':ab,ti,ca OR Bosnia:ab,ti,ca OR Herzegovina:ab,ti,ca OR 'Botswana':ab,ti,ca OR 'Brazil':ab,ti,ca OR 'Bulgaria':ab,ti,ca OR 'China':ab,ti,ca OR 'Colombia':ab,ti,ca OR 'Costa Rica':ab,ti,ca OR 'Cuba':ab,ti,ca OR 'Dominica':ab,ti,ca OR 'Dominican Republic':ab,ti,ca OR 'Ecuador':ab,ti,ca OR 'Equatorial Guinea':ab,ti,ca OR 'Fiji':ab,ti,ca OR 'Gabon':ab,ti,ca OR 'Grenada':ab,ti,ca OR 'Iran':ab,ti,ca OR 'Iraq':ab,ti,ca OR 'Jamaica':ab,ti,ca OR 'Jordan':ab,ti,ca OR 'Kazakhstan':ab,ti,ca OR 'Lebanon':ab,ti,ca OR 'Libya':ab,ti,ca OR 'Macedonia':ab,ti,ca OR 'Malaysia':ab,ti,ca OR 'Indian Ocean Islands':ab,ti,ca OR 'Maldives':ab,ti,ca OR 'Marshall Islands':ab,ti,ca OR 'Mauritius':ab,ti,ca OR 'Mexico':ab,ti,ca OR 'Montenegro':ab,ti,ca OR 'Namibia':ab,ti,ca OR 'Palau':ab,ti,ca OR 'Panama':ab,ti,ca OR 'Peru':ab,ti,ca OR 'Romania':ab,ti,ca OR 'Russia':ab,ti,ca OR 'Russian Federation':ab,ti,ca OR 'Serbia':ab,ti,ca OR 'Seychelles':ab,ti,ca OR 'South Africa':ab,ti,ca OR 'Saint Lucia':ab,ti,ca OR 'Saint Vincent and the Grenadines':ab,ti,ca OR 'Suriname':ab,ti,ca OR 'Thailand':ab,ti,ca OR 'Tonga':ab,ti,ca OR 'Tunisia':ab,ti,ca OR 'Turkey':ab,ti,ca OR 'Turkmenistan':ab,ti,ca OR 'Tuvalu':ab,ti,ca OR 'Venezuela':ab,ti,ca OR 'low resource':ab,ti,ca OR 'under resourced':ab,ti,ca OR 'resource poor':ab,ti,ca OR 'under developed':ab,ti,ca OR 'underdeveloped':ab,ti,ca OR 'developing country':ab,ti,ca OR 'developing countries':ab,ti,ca OR 'developing world':ab,ti,ca OR 'third world':ab,ti,ca OR lmic:ab,ti,ca OR (low:ab,ti,ca AND middle:ab,ti,ca AND income:ab,ti,ca) |
| 4 | #1 AND #2 AND #3 |
| 5 | #4 AND [embase]/lim NOT [medline]/lim  Limits: published 1992-present |

**3C: Web of Science**

| **Set #** | **Search terms** |
| --- | --- |
| 1 | cellphone OR cellphones OR mobile OR mobiles OR smartphone OR smartphones OR telephone OR telephones OR phone OR phones OR "e-health" OR ehealth OR "m-health" OR mhealth OR "automated telephone" OR IVR OR "interactive voice response" OR "voice message" OR "voice messages" OR voicemail OR "voice mail" OR sms OR text OR texts OR mms OR blackberry OR pda OR pdas OR ipad OR ipads OR android OR laptop OR laptops OR ((tablet OR tablets) AND (computer OR computers OR device OR devices)) |
| 2 | cervix OR cervices OR cervical OR colposcopy OR colposcopies OR colposcope OR colposcopes OR cervicography OR "pap test" OR "pap tests" OR "pap smear" OR "pap smears" OR "visual inspection with acetic acid" OR papillomavirus OR HPV |
| 3 | 'Afghanistan' OR 'Bangladesh' OR 'Benin' OR 'Burkina Faso' OR 'Burundi' OR 'Cambodia' OR 'cabo verde' OR 'Central African Republic' OR 'Chad' OR 'Comoros' OR 'Congo' OR 'Eritrea' OR 'Ethiopia' OR 'Gambia' OR 'Guinea' OR 'Haiti' OR 'Kenya' OR 'Korea' OR 'Liberia' OR 'Madagascar' OR 'Malawi' OR 'Mali' OR 'Mozambique' OR 'Myanmar' OR 'Nepal' OR 'Niger' OR 'Rwanda' OR 'Sierra Leone' OR 'Somalia' OR 'Tajikistan' OR 'Tanzania' OR 'Togo' OR 'Uganda' OR 'Zimbabwe' OR 'Armenia' OR 'Bhutan' OR 'Bolivia' OR 'Cameroon' OR 'Cape Verde' OR 'Congo' OR 'Cote dIvoire' OR 'ivory coast' OR 'Djibouti' OR 'Egypt' OR 'El Salvador' OR 'Georgia' OR 'Ghana' OR 'Guatemala' OR 'Guyana' OR 'Honduras' OR 'Indonesia' OR 'India' OR 'Kiribati' OR 'Kosovo' OR 'Kyrgyzstan' OR 'Kyrgyz' OR 'Laos' OR 'lao' OR 'Lesotho' OR 'Mauritania' OR 'Micronesia' OR 'Moldova' OR 'Mongolia' OR 'Morocco' OR 'Nicaragua' OR 'Nigeria' OR 'Pakistan' OR 'Papua New Guinea' OR 'Paraguay' OR 'Philippines' OR 'Samoa' OR 'Atlantic Islands' OR 'Sao Tome' OR Principe OR 'Senegal' OR 'Melanesia' OR 'Solomon islands' OR 'Sri Lanka' OR 'Sudan' OR 'Swaziland' OR 'Syria' OR 'East Timor' OR 'Timor leste' OR 'Ukraine' OR 'Uzbekistan' OR 'Vanuatu' OR 'Vietnam' OR 'Middle East' OR 'west bank' OR 'Gaza' OR 'Yemen' OR 'Zambia' OR 'Angola' OR 'Albania' OR 'Algeria' OR 'Argentina' OR 'Samoa' OR 'Azerbaijan' OR 'Republic of Belarus' OR 'Belize' OR Bosnia OR Herzegovina OR 'Botswana' OR 'Brazil' OR 'Bulgaria' OR 'China' OR 'Colombia' OR 'Costa Rica' OR 'Cuba' OR 'Dominica' OR 'Dominican Republic' OR 'Ecuador' OR 'Equatorial Guinea' OR 'Fiji' OR 'Gabon' OR 'Grenada' OR 'Iran' OR 'Iraq' OR 'Jamaica' OR 'Jordan' OR 'Kazakhstan' OR 'Lebanon' OR 'Libya' OR 'Macedonia' OR 'Malaysia' OR 'Indian Ocean Islands' OR 'Maldives' OR 'Marshall Islands' OR 'Mauritius' OR 'Mexico' OR 'Montenegro' OR 'Namibia' OR 'Palau' OR 'Panama' OR 'Peru' OR 'Romania' OR 'Russia' OR 'Russian Federation' OR 'Serbia' OR 'Seychelles' OR 'South Africa' OR 'Saint Lucia' OR 'Saint Vincent and the Grenadines' OR 'Suriname' OR 'Thailand' OR 'Tonga' OR 'Tunisia' OR 'Turkey' OR 'Turkmenistan' OR 'Tuvalu' OR 'Venezuela' OR 'low resource' OR 'under resourced' OR 'resource poor' OR 'under developed' OR 'underdeveloped' OR 'developing country' OR 'developing countries' OR 'developing world' OR 'third world' OR lmic OR (low AND middle AND income) |
| 4 | #1 AND #2 AND #3 |
| 5 | #4, Limits: published 1992-present |

**3D: Scopus**

| **Set #** | **Search terms** |
| --- | --- |
| 1 | TITLE-ABS-KEY ( cellphone OR cellphones OR mobile OR mobiles OR smartphone OR smartphones OR telephone OR telephones OR phone OR phones OR "e-health" OR ehealth OR "m-health" OR mhealth OR "automated telephone" OR ivr OR "interactive voice response" OR "voice message" OR "voice messages" OR voicemail OR "voice mail" OR sms OR "text messages" OR "text messages" OR texts OR blackberry OR pda OR pdas OR ipad OR ipads OR android OR laptop OR laptops OR ( ( tablet OR tablets ) AND ( computer OR computers OR device OR devices ) ) ) |
| 2 | TITLE-ABS-KEY (cervix OR cervices OR cervical OR colposcopy OR colposcopies OR colposcope OR colposcopes OR cervicography OR "pap test" OR "pap tests" OR "pap smear" OR "pap smears" OR "visual inspection with acetic acid" OR papillomavirus OR HPV) |
| 3 | TITLE-ABS-KEY ("Afghanistan" OR "Bangladesh" OR "Benin" OR "Burkina Faso" OR "Burundi" OR "Cambodia" OR "cabo verde" OR "Central African Republic" OR "Chad" OR "Comoros" OR "Congo" OR "Eritrea" OR "Ethiopia" OR "Gambia" OR "Guinea" OR "Haiti" OR "Kenya" OR "Korea" OR "Liberia" OR "Madagascar" OR "Malawi" OR "Mali" OR "Mozambique" OR "Myanmar" OR "Nepal" OR "Niger" OR "Rwanda" OR "Sierra Leone" OR "Somalia" OR "Tajikistan" OR "Tanzania" OR "Togo" OR "Uganda" OR "Zimbabwe" OR "Armenia" OR "Bhutan" OR "Bolivia" OR "Cameroon" OR "Cape Verde" OR "Congo" OR "Cote dIvoire" OR "ivory coast" OR "Djibouti" OR "Egypt" OR "El Salvador" OR "Georgia" OR "Ghana" OR "Guatemala" OR "Guyana" OR "Honduras" OR "Indonesia" OR "India" OR "Kiribati" OR "Kosovo" OR "Kyrgyzstan" OR "Kyrgyz" OR "Laos" OR "lao" OR "Lesotho" OR "Mauritania" OR "Micronesia" OR "Moldova" OR "Mongolia" OR "Morocco" OR "Nicaragua" OR "Nigeria" OR "Pakistan" OR "Papua New Guinea" OR "Paraguay" OR "Philippines" OR "Samoa" OR "Atlantic Islands" OR "Sao Tome" OR Principe OR "Senegal" OR "Melanesia" OR "Solomon islands" OR "Sri Lanka" OR "Sudan" OR "Swaziland" OR "Syria" OR "East Timor" OR "Timor leste" OR "Ukraine" OR "Uzbekistan" OR "Vanuatu" OR "Vietnam" OR "Middle East" OR "west bank" OR "Gaza" OR "Yemen" OR "Zambia" OR "Angola" OR "Albania" OR "Algeria" OR "Argentina" OR "Samoa" OR "Azerbaijan" OR "Republic of Belarus" OR "Belize" OR Bosnia OR Herzegovina OR "Botswana" OR "Brazil" OR "Bulgaria" OR "China" OR "Colombia" OR "Costa Rica" OR "Cuba" OR "Dominica" OR "Dominican Republic" OR "Ecuador" OR "Equatorial Guinea" OR "Fiji" OR "Gabon" OR "Grenada" OR "Iran" OR "Iraq" OR "Jamaica" OR "Jordan" OR "Kazakhstan" OR "Lebanon" OR "Libya" OR "Macedonia" OR "Malaysia" OR "Indian Ocean Islands" OR "Maldives" OR "Marshall Islands" OR "Mauritius" OR "Mexico" OR "Montenegro" OR "Namibia" OR "Palau" OR "Panama" OR "Peru" OR "Romania" OR "Russia" OR "Russian Federation" OR "Serbia" OR "Seychelles" OR "South Africa" OR "Saint Lucia" OR "Saint Vincent and the Grenadines" OR "Suriname" OR "Thailand" OR "Tonga" OR "Tunisia" OR "Turkey" OR "Turkmenistan" OR "Tuvalu" OR "Venezuela" OR "low resource" OR "under resourced" OR "resource poor" OR "under developed" OR "underdeveloped" OR "developing country" OR "developing countries" OR "developing world" OR "third world" OR lmic OR (low AND middle AND income)) |
| 4 | #1 AND #2 AND #3 |
| 5 | #4, Limits: English, published 1992-present |

**3E: CINAHL**

| **Set #** | **Search terms** |
| --- | --- |
| 1 | (MH "Mobile Applications") OR (MH "Cellular Phone+") OR (MH "Text Messaging") OR (MH "Computers, Hand-Held+") OR (MH "Telemedicine+") OR TI(cellphone OR cellphones OR mobile OR mobiles OR smartphone OR smartphones OR telephone OR telephones OR phone OR phones OR "e-health" OR ehealth OR "m-health" OR mhealth OR "automated telephone" OR ivr OR "interactive voice response" OR "voice message" OR "voice messages" OR voicemail OR "voice mail" OR sms OR text OR texts OR mms OR blackberry OR pda OR pdas OR ipad OR ipads OR android OR laptop OR laptops OR ((tablet OR tablets) AND (computer OR computers OR device OR devices))) OR AB (cellphone OR cellphones OR mobile OR mobiles OR smartphone OR smartphones OR telephone OR telephones OR phone OR phones OR "e-health" OR ehealth OR "m-health" OR mhealth OR "automated telephone" OR ivr OR "interactive voice response" OR "voice message" OR "voice messages" OR voicemail OR "voice mail" OR sms OR text OR texts OR mms OR blackberry OR pda OR pdas OR ipad OR ipads OR android OR laptop OR laptops OR ((tablet OR tablets) AND (computer OR computers OR device OR devices))) |
| 2 | (MH "Papillomavirus Infections+") OR (MH "Papillomaviruses") OR (MH "Cervix Neoplasms+") OR (MH "Cervical Smears+") OR (MH "Cervix") OR (MH "Vagina") OR TI(cervix OR cervices OR cervical OR colposcopy OR colposcopies OR colposcope OR colposcopes OR cervicography OR "pap test" OR "pap tests" OR "pap smear" OR "pap smears" OR "visual inspection with acetic acid" OR papillomavirus OR HPV) OR AB(cervix OR cervices OR cervical OR colposcopy OR colposcopies OR colposcope OR colposcopes OR cervicography OR "pap test" OR "pap tests" OR "pap smear" OR "pap smears" OR "visual inspection with acetic acid" OR papillomavirus OR HPV) |
| 3 | (MH "Developing Countries") OR (MH "Low and Middle Income Countries")  OR TI ( 'Afghanistan' OR 'Bangladesh' OR 'Benin' OR 'Burkina Faso' OR 'Burundi' OR 'Cambodia' OR 'cabo verde' OR 'Central African Republic' OR 'Chad' OR 'Comoros' OR 'Congo' OR 'Eritrea' OR 'Ethiopia' OR 'Gambia' OR 'Guinea' OR 'Haiti' OR 'Kenya' OR 'Korea' OR 'Liberia' OR 'Madagascar' OR 'Malawi' OR 'Mali' OR 'Mozambique' OR 'Myanmar' OR 'Nepal' OR 'Niger' OR 'Rwanda' OR 'Sierra Leone' OR 'Somalia' OR 'Tajikistan' OR 'Tanzania' OR 'Togo' OR 'Uganda' OR 'Zimbabwe' OR 'Armenia' OR 'Bhutan' OR 'Bolivia' OR 'Cameroon' OR 'Cape Verde' OR 'Congo' OR 'Cote dIvoire' OR 'ivory coast' OR 'Djibouti' OR 'Egypt' OR 'El Salvador' OR 'Georgia' OR 'Ghana' OR 'Guatemala' OR 'Guyana' OR 'Honduras' OR 'Indonesia' OR 'India' OR 'Kiribati' OR 'Kosovo' OR 'Kyrgyzstan' OR 'Kyrgyz' OR 'Laos' OR 'lao' OR 'Lesotho' OR 'Mauritania' OR 'Micronesia' OR 'Moldova' OR 'Mongolia' OR 'Morocco' OR 'Nicaragua' OR 'Nigeria' OR 'Pakistan' OR 'Papua New Guinea' OR 'Paraguay' OR 'Philippines' OR 'Samoa' OR 'Atlantic Islands' OR 'Sao Tome' OR Principe OR 'Senegal' OR 'Melanesia' OR 'Solomon islands' OR 'Sri Lanka' OR 'Sudan' OR 'Swaziland' OR 'Syria' OR 'East Timor' OR 'Timor leste' OR 'Ukraine' OR 'Uzbekistan' OR 'Vanuatu' OR 'Vietnam' OR 'Middle East' OR 'west bank' OR 'Gaza' OR 'Yemen' OR 'Zambia' OR 'Angola' OR 'Albania' OR 'Algeria' OR 'Argentina' OR 'Samoa' OR 'Azerbaijan' OR 'Republic of Belarus' OR 'Belize' OR Bosnia OR Herzegovina OR 'Botswana' OR 'Brazil' OR 'Bulgaria' OR 'China' OR 'Colombia' OR 'Costa Rica' OR 'Cuba' OR 'Dominica' OR 'Dominican Republic' OR 'Ecuador' OR 'Equatorial Guinea' OR 'Fiji' OR 'Gabon' OR 'Grenada' OR 'Iran' OR 'Iraq' OR 'Jamaica' OR 'Jordan' OR 'Kazakhstan' OR 'Lebanon' OR 'Libya' OR 'Macedonia' OR 'Malaysia' OR 'Indian Ocean Islands' OR 'Maldives' OR 'Marshall Islands' OR 'Mauritius' OR 'Mexico' OR 'Montenegro' OR 'Namibia' OR 'Palau' OR 'Panama' OR 'Peru' OR 'Romania' OR 'Russia' OR 'Russian Federation' OR 'Serbia' OR 'Seychelles' OR 'South Africa' OR 'Saint Lucia' OR 'Saint Vincent and the Grenadines' OR 'Suriname' OR 'Thailand' OR 'Tonga' OR 'Tunisia' OR 'Turkey' OR 'Turkmenistan' OR 'Tuvalu' OR 'Venezuela' OR 'low resource' OR 'under resourced' OR 'resource poor' OR 'under developed' OR 'underdeveloped' OR 'developing country' OR 'developing countries' OR 'developing world' OR 'third world' OR lmic OR (low AND middle AND income) ) OR AB ( 'Afghanistan' OR 'Bangladesh' OR 'Benin' OR 'Burkina Faso' OR 'Burundi' OR 'Cambodia' OR 'cabo verde' OR 'Central African Republic' OR 'Chad' OR 'Comoros' OR 'Congo' OR 'Eritrea' OR 'Ethiopia' OR 'Gambia' OR 'Guinea' OR 'Haiti' OR 'Kenya' OR 'Korea' OR 'Liberia' OR 'Madagascar' OR 'Malawi' OR 'Mali' OR 'Mozambique' OR 'Myanmar' OR 'Nepal' OR 'Niger' OR 'Rwanda' OR 'Sierra Leone' OR 'Somalia' OR 'Tajikistan' OR 'Tanzania' OR 'Togo' OR 'Uganda' OR 'Zimbabwe' OR 'Armenia' OR 'Bhutan' OR 'Bolivia' OR 'Cameroon' OR 'Cape Verde' OR 'Congo' OR 'Cote dIvoire' OR 'ivory coast' OR 'Djibouti' OR 'Egypt' OR 'El Salvador' OR 'Georgia' OR 'Ghana' OR 'Guatemala' OR 'Guyana' OR 'Honduras' OR 'Indonesia' OR 'India' OR 'Kiribati' OR 'Kosovo' OR 'Kyrgyzstan' OR 'Kyrgyz' OR 'Laos' OR 'lao' OR 'Lesotho' OR 'Mauritania' OR 'Micronesia' OR 'Moldova' OR 'Mongolia' OR 'Morocco' OR 'Nicaragua' OR 'Nigeria' OR 'Pakistan' OR 'Papua New Guinea' OR 'Paraguay' OR 'Philippines' OR 'Samoa' OR 'Atlantic Islands' OR 'Sao Tome' OR Principe OR 'Senegal' OR 'Melanesia' OR 'Solomon islands' OR 'Sri Lanka' OR 'Sudan' OR 'Swaziland' OR 'Syria' OR 'East Timor' OR 'Timor leste' OR 'Ukraine' OR 'Uzbekistan' OR 'Vanuatu' OR 'Vietnam' OR 'Middle East' OR 'west bank' OR 'Gaza' OR 'Yemen' OR 'Zambia' OR 'Angola' OR 'Albania' OR 'Algeria' OR 'Argentina' OR 'Samoa' OR 'Azerbaijan' OR 'Republic of Belarus' OR 'Belize' OR Bosnia OR Herzegovina OR 'Botswana' OR 'Brazil' OR 'Bulgaria' OR 'China' OR 'Colombia' OR 'Costa Rica' OR 'Cuba' OR 'Dominica' OR 'Dominican Republic' OR 'Ecuador' OR 'Equatorial Guinea' OR 'Fiji' OR 'Gabon' OR 'Grenada' OR 'Iran' OR 'Iraq' OR 'Jamaica' OR 'Jordan' OR 'Kazakhstan' OR 'Lebanon' OR 'Libya' OR 'Macedonia' OR 'Malaysia' OR 'Indian Ocean Islands' OR 'Maldives' OR 'Marshall Islands' OR 'Mauritius' OR 'Mexico' OR 'Montenegro' OR 'Namibia' OR 'Palau' OR 'Panama' OR 'Peru' OR 'Romania' OR 'Russia' OR 'Russian Federation' OR 'Serbia' OR 'Seychelles' OR 'South Africa' OR 'Saint Lucia' OR 'Saint Vincent and the Grenadines' OR 'Suriname' OR 'Thailand' OR 'Tonga' OR 'Tunisia' OR 'Turkey' OR 'Turkmenistan' OR 'Tuvalu' OR 'Venezuela' OR 'low resource' OR 'under resourced' OR 'resource poor' OR 'under developed' OR 'underdeveloped' OR 'developing country' OR 'developing countries' OR 'developing world' OR 'third world' OR lmic OR (low AND middle AND income)) |
| 4 | #1 AND #2 AND #3 |
| 5 | #4, Limits: published 1992-present |

**3F. Google**

| Set # | Search terms |
| --- | --- |
| 1 | (“mhealth” OR “cell phone” or “technology”)AND (“developing country” OR “world”) AND(“cervical cancer screening” OR “pap test” OR “cervix”) .pdf |
| 2 | Took first 100 sources |
