# Supplementary material for: Digital Health Strategies for Cervical Cancer Control in Low- and Middle-Income Countries: Systematic Review of Current Implementations and Gaps in Research
Source: J Med Internet Res. 2021 May 27;23(5):e23350. doi: 10.2196/23350 (PMC8193495; doi:10.2196/23350)
Supplement: Multimedia Appendix 4 [file jmir_v23i5e23350_app4.docx]

**Multimedia Appendix 4: Characteristics of included studies**

| **First Author, Year** | **Study Setting & Design** | **Digital Health Strategy** | **Parallel Control Group (Yes/No)** | **Participants** | **Measured Outcomes** |  |
| --- | --- | --- | --- | --- | --- | --- |
| Asgary 2016^a^ | Ghana, Cross-sectional study | Community health nurses (CHNs) received 2-week on-site training initially to perform and analyze VIA and digital cervicography using a smartphone. Over a period of 3 months, the CHNs sent cervical images, diagnosis, and plan of care via SMS to an expert VIA reviewer and received text-message feedback within 24 hours. | No | - CHNs (n=15) - Women (n=169) - Expert reviewer (1) | - Agreement or disagreement of the diagnoses made with VIA plus cervicography by the CHNs with those made by the expert reviewer. |  |
| Asgary 2019^a^ | Ghana, Qualitative study | Same as Asgary 2016. | No | - CHNs (n=15) - Women (n=21) - Nurse supervisor and the expert reviewer (n=2) | - Qualitative descriptions of acceptability and feasibility of smartphone-based training of CHNs |  |
| Bhatt 2018 | India, pilot study with qualitative component | Trained nurses conducted VIA screening, recorded data using forms in a SIM-based application loaded on a feature phone, and uploaded data using SMS. | No | - Participants in cervical cancer screening activities only^b^ - Nurses (n=9) - Women (n=170) | - Usage of mHealth prototype (measured by number of forms submitted) - Attendance at follow up appointment by VIA positive women. - Post- 6 month qualitative feedback on experience with prototype. |  |
| Caster 2017 | Malawi, pre-post study | Participants received a 30 minute tablet-based interactive education intervention comprising video content on cervical cancer. | No^c^ | - Women (n=243) | - The pretest-posttest group knowledge improvement score and post-test only group mean posttest score - Acceptability and feasibility of tablet-based lesson. |  |
| Catarino 2015 | Madagascar, cross-sectional study | Smartphone digital VIA images were taken by a medical student. Telemedicine was used to compare the on-site physician diagnosis to the off-site physician diagnosis based on digital VIA images. | No | - Women (n=332) | - Diagnostic reliability (sensitivity and specificity of diagnosis) using digital VIA images by the on-site physician and the off-site expert. - Inter-observer agreement between on-site physician and off-site expert diagnosis based on digital VIA images. |  |
| Devi 2018 | India, one- group pretest posttest design | Women attending out-patient services at the gynecology department received an educational booklet and invitation to participate in a screening program. Women who did not present for screening received reminders via letters, telephone calls or via SMS. | No | - Women (n=167) | - Participation in cervical cancer screening after reminder (by reminder type) |  |
| Erwin 2019 | Tanzania, randomized, double-blind, controlled trial | Women were randomly allocated to receive either (1) 15 behavior change communication messages via SMS alone, (2) 15 BCC SMS + a transportation eVoucher, or (3) a single SMS informing about the nearest cervical cancer screening clinic (control group). | Yes | - Women (n=863) | - Attendance at cervical cancer screening within 60 days of receiving the intervention or control messages. |  |
| Gallay 2017 | Madagascar, cross-sectional study | HPV-positive women were screened using an android “Exam” app to acquire cervical images. Images were uploaded to a databased and reviewed by expert gynecologists for image quality. | No | - Women (n=56) - Expert gynecologists (n=3) | - Photo quality evaluation - Feasibility of application |  |
| Huchko 2019^d^ | Kenya, mixed methods study within a cluster-randomized trial | Women undergoing HPV-based cervical cancer screening could opt to receive the results via text message, cell phone call, during a clinic visit, or via home visit. | Yes | - Women (n=4,947) | - Proportion of women successfully receiving the notification by notification type. - Time to treatment among HPV-positive women - Relationship between notification type and treatment uptake among HPV-positive women. |  |
| Khademolhosseini 2017 | Iran, randomized controlled trial^e^ | Patients received text messages, electronic posters, infographics, podcasts, and video tutorials through Telegram, an instant messaging service, providing education about Pap smear tests. | Yes | - Women (n=106) | - Mean pre-post difference in knowledge and items related to Health Belief Model constructs in the intervention versus control group measured immediately and 3 months post intervention. |  |
| Lima 2017 | Brazil, pretest, posttest study with random allocation | Women were randomly assigned to receive one of two interventions: (1) cervical cancer education and scheduling of screening exam (educational intervention) or (2) a reminder and scheduling of screening exam (behavioral intervention. Both interventions were delivered by telephone (either via landline or mobile phone) | No | - Women (n=524) | - A comparison of pre-test and post-test cervical cancer knowledge and attitude for women by intervention group - Rates of screening uptake by intervention group |  |
| Linde 2020 | Tanzania, randomized controlled trial | HPV-positive women were randomly assigned to one of two groups: standard care, a written appointment card with followup appointment information, or standard care plus text messages with educative messages or reminders | Yes | - Women (n=705) | - Attendance rate of follow-up cervical cancer screening among HPV-positive women by intervention group |  |
| Littman-Quinn 2013 | Botswana, description of program implementation | Clinicians used a smartphone to acquire cervical images and relevant medical data. These images and relevant medical data were sent via mobile phone to an in-country remote specialist, and if needed, to an international specialist. | No | - Women (n=356) - Clinicians (n=2) | - No quantitative outcomes presented |  |
| Ndlovu 2014 | Botswana, description of program scale-up | Same as Littman-Quinn 2013 except the ClickDiagnostics software used to capture patient data was replaced by Open Data Kit (ODK) software | No | - Ns not reported | - No quantitative outcomes presented |  |
| Parham 2010 | Zambia, Description of program implementation | Nurses uploaded patient information and cervical images to a consultation website. An automatic SMS was sent to the experts, including the “on call” consultant to expedite the review of the image. The experts reviewed the image and texted or called back the nurse back with the diagnosis. | No | - Nurses from 18 clinics (Ns not reported) | - No quantitative outcomes presented |  |
| Peterson 2016 | Kenya, Description of program implementation | Nurses used the Eva system (a low cost-colposcope built around a smartphone) to screen women. | No | - Women (n=824) | - Number of women screened with the Eva system - Number of women treated with cryotherapy |  |
| Quercia 2018 | Madagascar, cross-sectional study | Medical staff used an android-based Cervical Cancer Prevention System application to collect data from cervical cancer patients. | No | - Women (n=151) | - Feasibility - Technical errors reported by system |  |
| Quinley 2011 | Botswana, cross-sectional study | Nurses took digital images of the cervix obtained using a mobile phone and shared them with an expert gynecologist for review via MMS. | No | - Women (n=95) - Nurse midwives (n=4) - Expert gynecologist (n=1) | - Diagnostic agreement between nurse midwives and expert gynecologist. - Percentage of cases where there was diagnostic agreement using VIA and digital images by nurses - Percentage of women for whom the digital images were of insufficient quality |  |
| Rashid 2013a^g^ | Malaysia, study protocol | Women were recalled for a repeat PAP smear using one of four methods: letter, registered letters, SMS, or phone call | Yes | - No participants enrolled since this is a study protocol. | No outcome data reported since this is a study protocol. |  |
| Rashid 2013b^g^ | Malaysia, randomized control trial | As described for Rashid 2013a | Yes | - Women (n=1000) | - Percentage of women who received the recall notice by recall method - Percentage of women who presented for repeat PAP smears by recall method |  |
| Rashid 2014^g^ | Malaysia, cost-effectiveness analysis | As described for Rashid 2013a | Yes | - As described for Rashid 2013b. | - Cost-effectiveness of PAP smear by recall method |  |
| Ricard-Gauthier 2015 | Madagascar, cross-sectional study | An on-site gynecologist captured pictures with a smartphone during the cervical exam. The images were sent to off-site experts for diagnosis on a computer screen then 3 months later, the original gynecologists reviewed the smartphone images on a tablet screen to compare diagnoses (smartphone images were not used in original diagnosis). | No | - Women (n=88) - Experts (n=4) - On-site gynecologist (n=1) | - Sensitivity and specificity for CIN2+ were compared between on-site and off-site observers, using histopathological diagnosis as the criterion standard |  |
| Romli 2020 | Malaysia, controlled community trial | All subjects received an educational talk on cervical cancer and cervical cancer screening and the intervention group received text messages in addition to the talk. Pre- and post-tests measured cervical cancer knowledge and attitude. Uptake of pap test was tracked as well. | Yes | - Women (n=210) | - Impact on pap test knowledge and attitude scores - Uptake of pap test |  |
| Sharma 2018 | India, cross-sectional study | A trained nurse used a smartphone to capture cervical images during VIA. Images were shared fortnightly with an expert gynecologist for assessment. | No | - Women (n=180) - Nurse (n=1) - Expert (n=1) | - Diagnostic agreement between nurse led VIA testing and expert review of smartphone images |  |
| Swanson 2018 | Kenya, descriptive study | Women undergoing HPV-based cervical cancer screening could opt to receive the results via text message, cell phone call, during a clinic visit, or via home visit. | No | - Women (n=255) | - Percentage of participants with successful notification stratified by notification method - Percentage of HPV+ participants presenting for treatment stratified by notification method |  |
| Taghavi 2018 | India, cross-sectional study | A smartphone-based colposcope (Gynocular colposcope attached to a smartphone) was used for live examination and to capture digital cervical images. The images were evaluated for CIN2+ lesions by the on-site colposcopist and by off-site experts. | No | - Women (n=94) - Colposcopist/senior gynecology oncologist(n=1) - Experts (n=6) | - Swede scores from the live exam by the colposcopist versus and assessment of digital images by experts were compared. |  |
| Tran 2018 | Madagascar, fully crossed multiple-reader multiple-case design | Health providers captured cervical images using a smartphone during VIA/VILI procedures and uploaded to an online database for review by off-site gynecologists. | No | - Women (n=125) - Off-site gynecologists (n=45) | - Smartphone-captured images were compared for sensitivity and specificity of gynecologist review. - Expert and novice gynecologists were also compared for sensitivity and specificity |  |
| Urner 2017 | Madagascar, prospective study | Health providers captured cervical images using a smartphone during VIA/VILI procedures and uploaded to an online database for review by off-site gynecologists. | No | - Women (n=120) - Clinicians (n=15) | - Smartphone-captured images were compared for sensitivity and specificity of expert review. - Expert and novice clinicians were also compared for sensitivity and specificity |  |
| Yeates 2016 | Tanzania, cross-sectional study | Non-physician health workers (nurses and assistant medical officers) used a smartphone camera to capture cervical images during VIA. The images, proposed diagnosis and treatment plan were shared with and expert reviewer via WhatsApp for feedback. | No | - Women (n=1,072) - Nurses (n=3) - Assistant medical officers (n=2) - Experts (n=3) | - Diagnostic agreement between non-physician health worker and expert review of digital images - Time to take images (minutes) - Time for expert feedback (minutes) |  |
| Yeates 2020 | Tanzania, cross-sectional study | Same as Yeates 2016 except using the SEVIA app | No | - Women (n=10,545) - Experts (n=16) | - Diagnostic agreement between non-physician health worker and expert review of digital images - Time to take images (minutes) - Time for expert feedback (minutes) |  |
| **Abbreviations:** BCC, Behavior Change Communication; CHN, Community Health Nurse; CIN2+, Cervical Intraepithelial Neoplasia grade 2+; HPV, Human Papillomavirus; MMS, Multimedia Messaging Service; Ns, Number of participants; SIM, Subscriber Identity Module; SMS, Short Message Service; VIA, Visual Inspection with Acetic Acid; VILI, Visual Inspection with Lugol’s Iodine.  **Footnotes:**  ^a^Records describe findings from the same study and were assessed cumulatively for risk of bias and outcomes.  ^b^The study also included screening for oral cancers. Only details relevant to cervical cancer screening are presented in the table. Breakdown between participants from oral cancer and cervical cancer screening activities not provided  ^c^No control group; pre-test served as individual control  ^d^The digital health strategy was not randomly assigned.  ^e^Authors described the study design as quasi-experimental. However, due to the random assignment of participants to the intervention or control group described in the methods section, the review authors classified the study as an RCT.  ^g^Records describe findings from the same study and were assessed cumulatively for risk of bias and outcomes. | | | | | | |
