# Supplementary material for: Digital Health Strategies for Cervical Cancer Control in Low- and Middle-Income Countries: Systematic Review of Current Implementations and Gaps in Research
Source: J Med Internet Res. 2021 May 27;23(5):e23350. doi: 10.2196/23350 (PMC8193495; doi:10.2196/23350)
Supplement: Multimedia Appendix 5 [file jmir_v23i5e23350_app5.docx]

**Multimedia Appendix 5: Quality of reporting of including studies using the mHealth evidence reporting and assessment (mERA) checklist**

| **mERA Checklist Items**^a^ | | | | | | | | | | | | | | | | | |
| --- | --- | --- | --- | --- | --- | --- | --- | --- | --- | --- | --- | --- | --- | --- | --- | --- | --- |
| **Reference** | **Infrastructure** | **Technology Platform** | **Interoperability** | **Intervention Delivery** | **Intervention Content** | **Usability Testing** | **User Feedback** | **Access of Individual Participants** | **Cost Assessment** | **Adoption Inputs/Program Entry** | **Limitations for Delivery at Scale** | **Contextual Adaptability** | **Replicability** | **Data Security** | **Compliance with National Guidelines or Regulatory Statues** | **Fidelity of the Intervention** | **Total mERA Score (Max=16)** |
| ^b^**Asgary 2016**  **Asgary 2019** | 0 | 1 | 1 | 1 | 0 | 0 | 1 | 0 | 0 | 1 | 1 | 0 | 0 | 1 | 1 | 0 | **8** |
| **Bhatt 2018** | 0 | 1 | 0 | 1 | 1 | 1 | 1 | 0 | 0 | 0 | 0 | 1 | 0 | 1 | 0 | 1 | **8** |
| **Caster 2015** | 0 | 1 | 0 | 1 | 1 | 0 | 1 | 0 | 0 | 1 | 0 | 0 | 0 | 0 | 1 | 0 | **6** |
| **Catarino 2015** | 0 | 1 | 0 | 1 | 0 | 0 | 0 | 0 | 1 | 1 | 0 | 0 | 0 | 0 | 1 | 0 | **5** |
| **Devi 2018** | 0 | 0 | 0 | 1 | 0 | 0 | 0 | 0 | 0 | 0 | 0 | 0 | 0 | 0 | 0 | 0 | **1** |
| **Erwin 2019** | 0 | 1 | 0 | 1 | 1 | 1 | 0 | 1 | 0 | 1 | 1 | 1 | 0 | 1 | 0 | 1 | **10** |
| **Gallay 2017** | 0 | 1 | 0 | 1 | 1 | 0 | 0 | 0 | 0 | 0 | 0 | 0 | 1 | 1 | 1 | 1 | **7** |
| **Huchko 2019** | 0 | 1 | 0 | 1 | 1 | 1 | 1 | 0 | 0 | 0 | 1 | 1 | 1 | 0 | 0 | 1 | **9** |
| **Khademolhosseini 2017** | 0 | 1 | 0 | 1 | 1 | 0 | 0 | 0 | 0 | 1 | 0 | 0 | 0 | 0 | 1 | 0 | **5** |
| **Lima 2017** | 0 | 0 | 0 | 1 | 1 | 0 | 0 | 0 | 0 | 0 | 0 | 0 | 0 | 0 | 1 | 0 | **3** |
| **Linde 2020** | 0 | 1 | 0 | 1 | 1 | 1 | 0 | 1 | 0 | 1 | 1 | 1 | 1 | 1 | 1 | 1 | **12** |
| **Littman-Quinn 2013** | 1 | 1 | 1 | 1 | 0 | 0 | 0 | 1 | 1 | 1 | 1 | 0 | 0 | 1 | 1 | 0 | **10** |
| **Ndlovu 2014** | 1 | 1 | 1 | 1 | 0 | 0 | 1 | 0 | 1 | 0 | 1 | 1 | 0 | 1 | 0 | 0 | **9** |
| **Parham 2010** | 0 | 1 | 0 | 1 | 0 | 0 | 0 | 0 | 1 | 1 | 0 | 0 | 0 | 1 | 0 | 0 | **5** |
| **Peterson 2016** | 0 | 1 | 0 | 1 | 0 | 0 | 0 | 0 | 1 | 1 | 0 | 0 | 0 | 1 | 0 | 0 | **5** |
| **Quercia 2018** | 1 | 1 | 1 | 1 | 1 | 0 | 1 | 0 | 0 | 0 | 0 | 1 | 0 | 1 | 0 | 1 | **9** |
| **Quinley 2011** | 0 | 1 | 0 | 1 | 0 | 0 | 0 | 0 | 0 | 1 | 0 | 0 | 0 | 0 | 1 | 0 | **4** |
| ^b^**Rashid 2013a**  **Rashid 2013b**  **Rashid 2014** | 0 | 1 | 0 | 1 | 1 | 0 | 1 | 1 | 1 | 0 | 0 | 0 | 0 | 1 | 1 | 1 | **9** |
| **Ricard-Gauthier 2015** | 0 | 1 | 0 | 1 | 0 | 0 | 0 | 0 | 0 | 0 | 0 | 0 | 0 | 1 | 1 | 0 | **4** |
| **Romli 2020** | 0 | 0 | 0 | 1 | 1 | 0 | 0 | 0 | 0 | 0 | 0 | 0 | 0 | 0 | 1 | 0 | **3** |
| **Sharma 2018** | 0 | 1 | 1 | 1 | 0 | 0 | 1 | 1 | 0 | 1 | 0 | 0 | 0 | 0 | 1 | 1 | **8** |
| **Swanson 2018** | 0 | 1 | 0 | 1 | 0 | 0 | 0 | 1 | 0 | 1 | 1 | 0 | 0 | 0 | 1 | 0 | **6** |
| **Taghavi 2018** | 0 | 1 | 0 | 1 | 0 | 0 | 0 | 0 | 0 | 0 | 0 | 0 | 0 | 0 | 1 | 0 | **3** |
| **Tran 2018** | 0 | 1 | 0 | 1 | 1 | 0 | 0 | 0 | 0 | 1 | 0 | 0 | 1 | 0 | 0 | 0 | **5** |
| **Urner 2017** | 0 | 1 | 0 | 1 | 0 | 0 | 0 | 0 | 0 | 0 | 0 | 0 | 0 | 0 | 1 | 0 | **3** |
| **Yeates 2016** | 1 | 1 | 0 | 1 | 0 | 1 | 0 | 1 | 0 | 1 | 0 | 1 | 0 | 1 | 1 | 1 | **10** |
| **Yeates 2020** | 0 | 0 | 1 | 1 | 1 | 0 | 0 | 0 | 1 | 1 | 1 | 0 | 1 | 1 | 1 | 1 | **10** |
| **Total number of studies describing mERA item** | **4** | **23** | **6** | **27** | **13** | **5** | **8** | **7** | **7** | **15** | **8** | **7** | **5** | **14** | **18** | **10** |  |
| **Footnotes:** ^a^A score of 0 indicates that the mERA item was not described, while a score of 1 means that the item was described. Maximum possible score for any study was 16.  ^b^For the studies by Asgary et. al and Rashid et. al, a cumulative mERA score was calculated from all included records. | | | | | | | | | | | | | | | | | |
